# The DNA-Binding Domain of Human PARP-1 Interacts with DNA Single-Strand Breaks as a Monomer through Its Second Zinc Finger

**DOI:** 10.1016/j.jmb.2011.01.034

**Published:** 2011-03-18

**Authors:** Sebastian Eustermann, Hortense Videler, Ji-Chun Yang, Paul T. Cole, Dominika Gruszka, Dmitry Veprintsev, David Neuhaus

**Affiliations:** MRC Laboratory of Molecular Biology, Hills Road, Cambridge CB2 0QH, UK

**Keywords:** poly(ADP ribose)polymerase-1, zinc finger, solution structure, DNA repair, DNA single-strand breaks, BER, base excision repair, BRCT, breast cancer susceptibility protein C-terminal domain, DL3, DNA ligase IIIα, EMSA, electromobility shift assay, F1, finger 1, F2, finger 2, HSQC, heteronuclear single-quantum coherence, NOE, nuclear Overhauser effect, NOESY, NOE spectroscopy, PAR, poly(ADP-ribose), PARP-1, poly(ADP-ribose)polymerase-1, RDC, residual dipolar coupling, SSBR, single-strand break repair, ssDNA, single-stranded DNA, SV-AUC, sedimentation velocity analytical ultracentrifugation

## Abstract

Poly(ADP-ribose)polymerase-1 (PARP-1) is a highly abundant chromatin-associated enzyme present in all higher eukaryotic cell nuclei, where it plays key roles in the maintenance of genomic integrity, chromatin remodeling and transcriptional control. It binds to DNA single- and double-strand breaks through an N-terminal region containing two zinc fingers, F1 and F2, following which its C-terminal catalytic domain becomes activated *via* an unknown mechanism, causing formation and addition of polyadenosine-ribose (PAR) to acceptor proteins including PARP-1 itself. Here, we report a biophysical and structural characterization of the F1 and F2 fingers of human PARP-1, both as independent fragments and in the context of the 24-kDa DNA-binding domain (F1 + F2). We show that the fingers are structurally independent in the absence of DNA and share a highly similar structural fold and dynamics. The F1 + F2 fragment recognizes DNA single-strand breaks as a monomer and in a single orientation. Using a combination of NMR spectroscopy and other biophysical techniques, we show that recognition is primarily achieved by F2, which binds the DNA in an essentially identical manner whether present in isolation or in the two-finger fragment. F2 interacts much more strongly with nicked or gapped DNA ligands than does F1, and we present a mutational study that suggests origins of this difference. Our data suggest that different DNA lesions are recognized by the DNA-binding domain of PARP-1 in a highly similar conformation, helping to rationalize how the full-length protein participates in multiple steps of DNA single-strand breakage and base excision repair.

## Graphical Abstract

**Figure:**
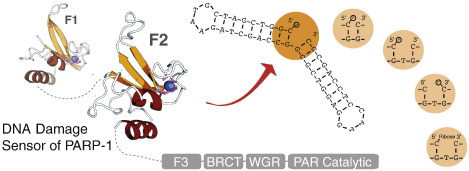

**Research Highlights:**

► PARP-1 zinc fingers 1 and 2 are structurally independent in the free state. ► PARP-1 fingers 1 + 2 form a 1:1 monomeric complex with DNA single-strand breaks. ► PARP-1 fingers 1 + 2 recognize different types of DNA single-strand break similarly. ► DNA damage recognition by PARP-1 occurs predominantly through finger 2.

## Introduction

PolyADP-ribosylation is a posttranslational modification in which poly(ADP-ribose) (PAR), a highly negatively charged, branched-chain polymer formed from ADP-ribose units derived from nicotinamide adenine dinucleotide, is attached to acceptor groups on proteins.^1^ This modification is carried out by poly(ADP-ribose)polymerase (PARP) enzymes, of which the first discovered and most important is PARP-1, a very highly abundant nuclear protein found in eukaryotes higher than *Saccharomyces.*^2^ The main targets for addition of PAR by PARP-1 are histones and PARP-1 itself,^3,4^ although there are many other targets including HMG proteins,^5^ topoisomerase I^6^ and p53.^7^

The biological functions of PARP-1 are not fully understood, but it is involved in a large number of fundamentally important cellular processes. It was discovered early that the rate of PAR synthesis by PARP-1 is greatly increased in response to DNA damage,^8^ and consequently, much of the literature on PARP-1 over the past several decades has been concerned with its role in DNA repair and maintenance of genome stability (reviewed in Refs. 9–12). Although PARP-1 does not itself participate directly in the chemical reactions that DNA undergoes during repair, many studies have shown that activation of PARP-1 following binding to DNA single-strand breaks is important in both base excision repair (BER) and single-strand break repair (SSBR) DNA repair processes (reviewed in Refs. 13 and 14). Several possible roles have been proposed for PARP-1 in these processes, including scanning for strand breaks, recruitment of other DNA repair proteins such as XRCC-1 (X-ray repair cross-complementing group 1) and DNA ligase III, decondensation of chromatin structure and protection of repair intermediates having single-strand breaks.^13^ In addition, PARP-1 is involved in detection and reinitiation of stalled DNA replication forks.^15^ Also, while much of the literature is concerned with binding to single-strand breaks, it is clear that PARP-1 binds to a number of other DNA structures, including double-strand breaks, cruciforms and hairpins,^16,17^ and there are reports of sequence-specific binding.^18,19^ More recently, it has become clear that PARP-1 is involved in many other cellular processes, not all of which apparently involve DNA-mediated activation of the catalytic domain at all.^20^ Following extensive DNA damage, PARP-1 plays a key role in determining cell fate between DNA repair and cell death, either through apoptosis or through necrosis.^12,21^ Although binding of PARP-1 to DNA single-strand breaks remains a matter of fundamental importance in understanding the functions of PARP-1, especially in relation to its roles in DNA repair, as yet no high-resolution structural information has been published concerning the way in which this recognition is achieved.

**Fig. 1:**
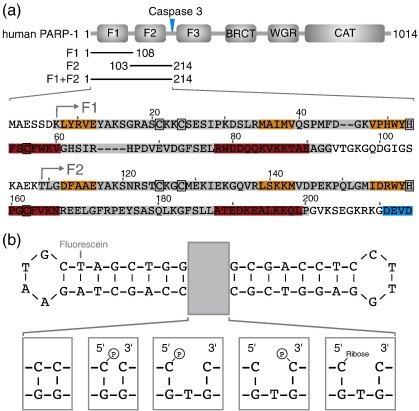
Protein and DNA constructs used in this study. (a) During apoptosis, PARP-1 is cleaved by caspase-3^22^ into a 24-kDa fragment that contains the two N-terminal zinc fingers (F1 and F2) and an 89-kDa fragment composed of the third zinc finger (F3), the BRCT domain, the WGR domain and the C-terminal catalytic domain (the caspase-3 cleavage site is marked with a blue arrow). The expansion below shows the sequence of human PARP-1 residues 1–214, highlighting the fragments used in this study: F1 (residues 1–108), F2 (residues 103–214) and F1 + F2 (residues 1–214). Secondary structural elements are colored (α-helices, red; β-strands, orange), the caspase-3 recognition site is shown in blue and zinc-coordinating residues are indicated by black boxes. (b) Synthetic DNA dumbbell ligands used in this study. The same dumbbell scaffold was used to harbour different types of DNA strand breaks: a 5′-phosphorylated nick, a 5′-phosphorylated single nucleotide gap, a 3′-phosphorylated single nucleotide gap and a 5′-ribosylated single nucleotide gap (which results from strand incision of an abasic site during BER). Ligation of the nicked DNA dumbbell ligand produced a circular DNA without a strand break (left-handmost box).

The domain structure of PARP-1 (Fig. 1) comprises an N-terminal DNA-binding domain that contains two homologous zinc fingers F1 and F2, a nuclear localization signal, a recently characterized domain containing a third zinc finger F3 that is unrelated to F1 and F2, not involved in DNA binding but essential for activation,^23,24^ a breast cancer susceptibility protein C-terminal (BRCT) domain involved in protein–protein interactions, a WGR domain and the catalytic domain. Recognition of DNA strand breaks occurs through the two N-terminal zinc fingers F1 and F2,^25^ without which PARP-1 is incapable of DNA-mediated activation. The two-finger fragment that we study here, PARP-1 1–214 (hereinafter referred to as F1 + F2), corresponds to the apoptotic fragment released through cleavage by caspase-3 and -7.^26,27^ This fragment contains the DNA-damage-binding activity relevant to activation, as shown by the observations that activation of full-length PARP-1 is suppressed *in vivo* by competitive binding of the F1 + F2 fragment to DNA damage sites during apoptosis or following overexpression^28,29^ and that the DNase I footprint of the F1 + F2 fragment on binding DNA harboring a single-strand break is identical to that of full-length PARP-1 (7 ± 1 nucleotides on each side of the break, independent of sequence context).^25^

The two N-terminal zinc fingers of PARP-1 are of a highly unusual type, characterized by a CCHC ligand pattern and a long sequence separation (26–37 residues) between ligands 2 and 3; other fingers of this family are found in DNA ligase IIIα (DL3), which has one, and the plant DNA 3′ phosphatase AtZDP, which has three.^30^ We previously determined the structure of the zinc finger from human DL3 (1uw0),^31^ since when structures of human PARP-1 F1 (2dmj) and F2 (2cs2) and *Arabidopsis thaliana* PARP-1 F1 (1v9x) have been deposited by the Riken Structural Genomics Initiative (RSGI), although in these later cases no related publications have appeared and the assignments and constraint data were not deposited. Mutagenesis results have been used to suggest that the two fingers of PARP-1 may have different roles despite their high similarity, but a clear overall picture from the literature is elusive. For instance, it has been suggested that PARP-1's principal DNA-binding activity may be contained in F2,^32^ while F1 is required to relay the signal for PAR formation to the catalytic domain,^33^ whereas another report suggests that both fingers are needed for activation by single-stranded nicks, but only F1 is needed for activation by double-stranded breaks.^34^ Thus, the differential role of the two fingers in the mechanism of DNA-damage recognition remains to be established.

Another important question concerning PARP-1 is how, upon binding of DNA strand breaks, the signal for activation is transmitted from the DNA-binding domain to the catalytic domain. A number of publications have pointed to possible roles for dimerization in this process. It has been established that automodification of PARP-1 is a bimolecular process,^35^ leading to the suggestion that the catalytic domain functions as a dimer. BRCT domains often function as protein–protein interaction domains and could promote dimerization for PARP-1. The third zinc finger domain F3 was initially reported to be dimeric on the basis of an extensive intermonomer interface found in its crystal structure, and a model for activation was proposed based on formation of this dimer.^23^ However, a subsequent study of F3 by NMR resulted in a monomeric solution structure having a different arrangement of the C-terminal helix,^24^ and the authors of the original crystal structure went on to show that mutations introduced into the dimer interface of F3 had no effect on activation (although mutations elsewhere in F3 did do so).^36^ It has also been reported that the DNA-binding domain of PARP-1 dimerizes upon binding to a 3′ DNA overhang.^37^ Overall, however, evidence that the process of activation itself necessarily involves dimerization of PARP-1 remains at best equivocal.

Here, we report a structural and biophysical characterization of the F1 and F2 zinc fingers of human PARP-1 and their interactions with DNA single-strand breaks, addressing the questions of what role these fingers play in DNA recognition and subsequent activation of PARP-1's catalytic activity. We show that in F1 + F2, the two zinc fingers are structurally independent in the absence of DNA. Using a combination of NMR spectroscopy, fluorescence measurements, analytical ultracentrifugation and electrophoretic gel mobility assays, we characterize the interaction of these fingers, both in the F1 + F2 fragment and as the individual F1 and F2 fingers, with model DNA ligands that represent species found during SSBR. The interaction of F1 + F2 with a DNA single-strand break appears to be essentially identical whether the break is a nick or a 1-nt gap; furthermore, we show that F1 + F2 binds as a monomer, contrary to much of what has been previously proposed in the literature. We show that F2 is primarily responsible for DNA-damage recognition, as it interacts very much more strongly with DNA single-strand breaks than does F1, and the mode of DNA binding for F2 appears to be identical whether it is studied in isolation or in the context of the two-finger F1 + F2 fragment. In addition, we present a mutational study that suggests the origins of the difference in DNA-binding affinities observed between F1 and F2 despite their homology.

## Results

### Structures of PARP-1 zinc fingers F1 and F2

Separate fragments corresponding to F1 (residues 1–108) and F2 (residues 103–214) of PARP-1 were cloned and overexpressed in *Escherichia coli* for structural analysis (Fig. 1a), selected on the basis of our previous sequence alignment amongst PARP-like zinc fingers from PARP-1, DL3 and AtZDP.^31^ The NMR signals of these fragments were assigned using a suite of triple-resonance multidimensional experiments, and structures of F1 and F2 were calculated from a combination of nuclear Overhauser effect (NOE) and torsion angle restraints. As shown by statistics in Table 1 (and the rmsd profiles in Supplementary Fig. 1a), the calculations resulted in structural ensembles that were well defined by the data. In each case, a small number of N- and C-terminal residues were disordered, as evidenced by the lack of any medium- or long-range NOE connectivities for these residues and by the sharpness and near-random-coil chemical shift values of their NMR resonances. Thus, the structured domains of the two fingers correspond to residues 7–93 (F1) and residues 109–200 (F2).

**Fig. 2:**
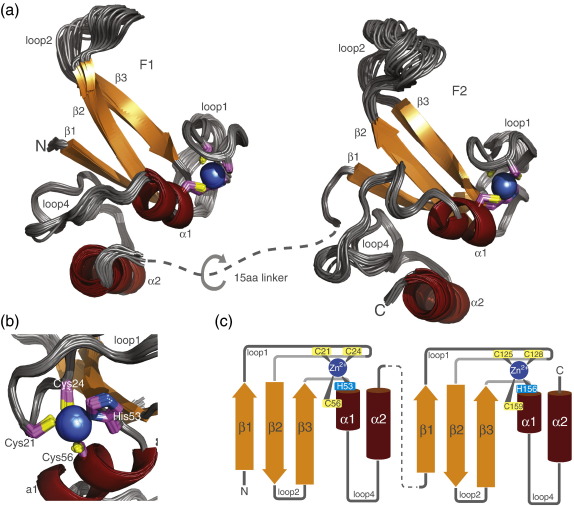
Solution structures of the N-terminal zinc fingers F1 and F2 of human PARP-1. (a) Ensemble views of the 30 accepted structures of F1 (backbone rmsd 0.39 Å) and F2 (backbone rmsd 0.37 Å) shown in a cartoon representation, colored as for Fig. 1. Zinc ions are shown as blue spheres and zinc coordinating residues in stick representation with carbon atoms in magenta. The two zinc fingers are connected by a linker of 15 residues (shown as a dotted line) that is flexible, as judged by NMR measurements of PARP-1 F1 + F2. (b) Close-up of the zinc coordination of PARP-1 F1 [rotated about the vertical by 90° relative to the view in (a)]. The absolute chirality of the zinc binding configuration is *S*, as defined by Berg.^38^ (c) Schematic showing the zinc binding and secondary-structure topology of the two N-terminal PARP-1 zinc finger domains F1 and F2, colored as for Fig. 1.

Ensemble views of the structures of F1 and F2 are shown in Fig. 2. Both fingers share the same overall architecture with the previously determined structure of the PARP-like zinc finger from DL3 (1uw0), comprising a three-stranded antiparallel β-sheet that carries the first pair of zinc-binding residues on an extended hairpin between strands 1 and 2, followed by two helices that pack together in a parallel fashion, the first of which carries the second pair of zinc-binding residues on its first turn. In F1, the sheet comprises strand 1 (residues 8–12), strand 2 (residues 35–39) and strand 3 (residues 48–52); helix 1 comprises residues 54–60; helix 2 comprises residues 78–92 and the metal binding residues are Cys21, Cys24, His53 and Cys56. Correspondingly, in F2 the sheet comprises strand 1 (residues 112–116), strand 2 (residues 138–143) and strand 3 (residues 154–158); helix 1 comprises residues 160–166; helix 2 comprises residues 188–199 and the metal binding residues are Cys125, Cys128, His159 and Cys162. In both fingers, both helices and the interhelix linker (loop 4) make extensive hydrophobic contacts to residues of the β-sheet. In addition, between helices 1 and 2, one residue (Asp72 in F1, Lys182 in F2) makes one or two hydrogen bonds to the edge of strand 1 of the β-sheet. However, this feature is probably on the borderline of detectability by NMR, because its formation depends on the precise arrangement between large-scale elements of the structure whose relationship is not highly constrained. Similar to the DL3 finger, both F1 and F2 of PARP-1 have an *S* absolute chirality of the zinc-binding ligands, as defined by Berg^38^ (see also Kulczyk *et al.*^31^), and the loops between strands 2 and 3 of the β-sheets are relatively disordered. These structures are similar to those previously deposited by RSGI for F1 (2dmj) and F2 (2cs2) of human PARP-1; for the ordered regions of F1 (residues 7–40 and 46–93) the backbone rmsd between our structure and 2dmj is 1.31 Å, while for the ordered regions of F2 (residues 109–144 and 152–200) the rmsd between our structure and 2cs2 is 1.37 Å (in each case, the ensemble member closest to the mean is used for the fitting).

The most significant difference between the structures of F1 and F2 lies in the path taken by the chain between helices 1 and 2 (Fig. 2a). In F2, there are four additional residues in this part of the sequence, and on exiting helix 1 the chain forms a further single turn of helix at almost 90° to the axis of helix 1; the shorter chain of F1 makes no such helical turn at this position, the chain instead takes a more direct route through a stretch of irregular structure toward the N-terminus of helix 2. Other differences between the structures of PARP-1 F1 and F2 comprise relatively small deviations in the path of the loops linking the β-strands, although mostly these are in parts of the structure that are in any case relatively flexible (see below); the largest such difference is that loop 2 (linking strands 2 and 3 of the sheet) is two residues longer in F2 than in F1. The overall backbone rmsd between corresponding ordered regions of F1 and F2 (fitting residues 7–40, 46–65 and 66–90 of F1 to residues 111–144, 152–171 and 176–200 of F2, taking in each case the ensemble member closest to the mean structure) is 2.33 Å (the corresponding statistic for similarly fitting 2dmj to 2cs2 is 2.38 Å).

### The F1 and F2 zinc finger domains of PARP-1 are structurally independent

The possibility of interfinger interactions involving F1 and F2 of PARP-1 is of particular interest given the probable differential roles of the fingers in PARP's function.

**Fig. 3:**
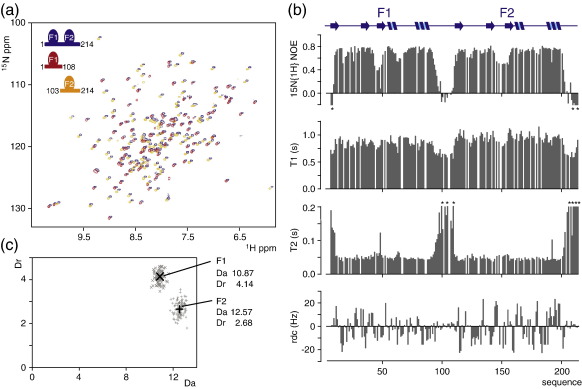
Fingers F1 and F2 of human PARP-1 are structurally independent. (a) Overlay of [^15^N–^1^H] HSQC spectra of PARP-1 fragments F1 (red peaks), F2 (yellow peaks) and F1 + F2 (blue peaks). To help visualize the relationships, we deliberately introduced a small systematic chemical shift offset for the spectrum of PARP-1 F1 + F2. (b) Internal motions of backbone amides of PARP-1 F1 + F2 were assessed by NMR spectroscopy. Steady-state ^15^N{^1^H} NOE values, ^15^N *T*_1_ and ^15^N *T*_2_ relaxation times and amide group RDC values are each plotted as a function of sequence. The relaxation data (upper three panels) demonstrate that the linker region between the two zinc fingers F1 and F2 (residues 94–108) is flexible, having internal motions that are faster than overall molecular tumbling; in addition, significant motions were detected for the protein termini and for some loop regions of each zinc finger (described in the main text). The RDC data (lowest panel) were recorded from a sample of PARP-1 F1 + F2 weakly aligned by pf1 phage, using [^15^N–^1^H] HSQC IPAP (in-phase antiphase) spectra.^39^ Larger values are only seen for the ordered regions. **(c)** Using the RDCs measured for PARP-1 F1 + F2 and the solution structure of the separated F1 and F2, we determined the axial (*D*_a_) and rhombic (*D*_r_) components of the alignment tensor of each finger in the context of a two-finger construct. Associated uncertainties were estimated using the program MODULE 2.0,^40^ setting the error of measurement for experimentally determined RDCs to 2 Hz (see Supplementary Material and Methods). The unrelated alignment tensors of F1 and F2 provide additional evidence for the lack of any rigid orientation between the two zinc fingers.

Several lines of evidence point to the conclusion that the two N-terminal zinc finger domains of PARP-1 are structurally independent in solution. The combined 24-kDa apoptotic fragment F1 + F2 comprising residues 1–214 was cloned and overexpressed in *E. coli*. Assignment of this fragment was relatively straightforward and was accomplished using experiments essentially identical to those used for the single finger fragments. Since the linewidths of signals from the F1 + F2 fragment were only slightly greater than those of the individual fingers, these experiments worked almost as well for the two-finger fragment as for the single fingers, and problems of overlap were greatly alleviated by comparing to the single-finger spectra where corresponding signals were extremely similar. Comparison of the (^15^N, ^1^H) heteronuclear single-quantum coherence (HSQC) spectra of the three fragments F1, F2 and F1 + F2 shows that apart from a very small number of residues immediately bordering the linker, the positions of signals from either of the ordered finger domains are completely unaffected by the presence or absence of the other finger (Fig. 3a). Given the well-known sensitivity of chemical shifts, especially those of amide groups in proteins, to even small environmental changes, this is in itself compelling evidence for structural independence of the fingers. In addition, careful analysis of NOE spectroscopy (NOESY) spectra from F1 + F2 failed to reveal any NOE connectivities linking the two fingers to one another.

To further reinforce the evidence for structural independence, we used measurements of RDCs to determine the relative alignment tensors of the two fingers seen when the fragment F1 + F2 was aligned by addition of pf1 phage. If the fingers were to interact to give a single globular structure, the measured RDC values from the ordered domain would all have to be consistent with a single alignment tensor, whereas if the fingers are independent, there would most probably be different alignment tensors for F1 and for F2. A similar approach to establishing interdomain independence was previously used by Braddock *et al.*^41^ Figure 3b shows the RDC data obtained for F1 + F2. It is apparent from the data that the magnitudes of the RDCs are systematically slightly larger for F2 than for F1, already suggesting that the domains have independent alignment tensors and that the strength of interaction with the phage is slightly stronger for the F2 domain than for F1. To confirm that the tensors for F1 and F2 differ significantly, we compared the best-fit tensor components found using the ISAC procedure of Sass *et al.*,^42^ as applied during independent structure calculations for F1 and for F2; for the lowest-energy structures of F1 and F2 these components are marked in Fig. 3c. We then used the program MODULE^40^ to estimate the reliability of these tensor components, using a Monte Carlo simulation to show the distribution of back-calculated tensor components expected from the structure, given the actual measurement error of ± 2 Hz in determination of the RDC values. Figure 3c shows that the distributions calculated for F1 and F2 form two separate clusters, confirming that the difference in alignment tensors between the fingers is real.

Figure 3b also shows the results of a ^15^N dynamics study of PARP-1 F1 and F2 in the context of F1 + F2. These data confirm the boundaries of the ordered domains and show very clearly that the linker region between the two fingers in the two-finger fragment is highly disordered, consistent with the lack of any defined mutual orientation between fingers as described above. In addition, regions of relatively high internal flexibility exist within each of the fingers. The most clearly defined of these is the β2–β3 loop (loop 2), for which the ^15^N{^1^H} NOE is markedly reduced in both F1 and F2, just as was previously observed for the corresponding region of the single finger of DL3.^31^ For F2, where loop 2 is two residues longer than in F1, this region of enhanced flexibility is slightly more extensive. For F1 there is also a small reduction of the ^15^N{^1^H} NOE just beyond the C-terminus of helix 1 (residues 62–66), indicating slightly enhanced flexibility in this region, although for the corresponding regions of F2 (and of the DL3 finger^31^) evidence for enhanced flexibility is lacking; F2 forms a helical turn in this region. We also examined the relaxation data for evidence concerning the possibility of any interfinger interaction. Interestingly, the data for the F1 and F2 regions show small systematic differences, especially in the *T*_1_ values, which is in itself a further indicator of their structural independence in the F1 + F2 fragment. The *T*_1_/*T*_2_ ratios for well-ordered residues (^15^N{^1^H} NOE > 0.65) in the two fingers F1 and F2 are approximately 18.4 and 21.6, respectively. Under the highly simplistic assumption of isotropic tumbling, these values might suggest tumbling that is somewhat slower than expected for the isolated individual fingers, but in reality this must be due at least in part to the fact that the two fingers are tethered together in the F1 + F2 fragment. This must impede significantly the tumbling of the individual fingers, in addition to probably making the tumbling highly anisotropic; a recent study of artificially linked GB1 domains by Walsh *et al*. has shown just such effects.^43^ These factors make it difficult to use the relaxation data to probe for possible interfinger interactions. We cannot rule out the possibility that a very weak, transient interaction might have some influence on the ^15^N relaxation data, but on balance, given also the unequivocal evidence described earlier from comparing RDC values, chemical shifts and NOEs that all point to the absence of any appreciable interaction, we conclude that there is most probably no interfinger interaction.

### PARP-1 F1 + F2 fragment recognizes DNA single-strand breaks as a monomer and in a 1:1 stoichiometry

To study binding of the zinc fingers of human PARP-1 to damaged DNA, we first used the nicked dumbbell DNA previously established as a ligand for the closely related single zinc finger of DL3.^31^ This comprises a single 44-nt DNA chain containing two self-complementary regions that anneal to form an 18-bp double helix with a single-stranded nick at the centre and a tetraloop at each end (Fig. 1). This design of ligand was chosen for a number of reasons. The base-paired region on each side of the nick is large enough to accommodate the 7-nt footprint of full-length PARP-1^25^ but within the context of a small, conformationally stable ligand. Also, it lacks double-stranded ends, which are known to bind to PARP-like zinc fingers, thereby allowing for assembly of a structurally homogeneous complex. The tetraloop sequences were selected because they form particularly stable turn conformations, thereby favoring the monomeric dumbbell form over polymeric alternatives. To maximize the similarity of our model ligand to actual intermediates occurring on DNA repair pathways, we used a dumbbell sequence that contained a terminal phosphate group on the 5′ side of the nick. Preliminary data (not shown) using other dumbbell ligands showed that, as expected, binding affinity is only very weakly affected by sequence variations within the stem regions (approximate binding affinities from electromobility shift assay (EMSA) experiments varied by less than a factor of 2 across the cases tested).

**Fig. 4:**
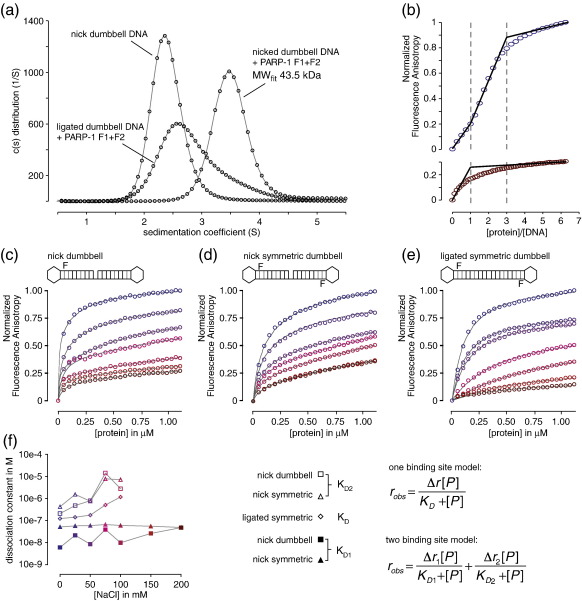
Biophysical characterization of PARP-1 binding to DNA single-strand breaks. (a) Sedimentation velocity experiments were carried out using 0.5 μM nicked dumbbell DNA alone or in the presence of a fivefold excess of PARP-1 F1 + F2. As a control, the latter experiment was repeated using a ligated version of the DNA dumbbell. Raw data were fitted as described in Supplementary Fig. 2, and diffusion-deconvoluted sedimentation coefficient distributions *c*(*s*) are plotted for each experiment. The *c*(*s*) distribution of the nicked dumbbell DNA + PARP-1 F1 + F2 sample corresponds to that expected for formation of a homogenous 1:1 protein–DNA complex (fitted molecular mass 43.5 kDa; calculated molecular mass 38 kDa). (b) Stochiometric fluorescence anisotropy titrations of 44 nt 5′-phosphorylated fluorescently labeled DNA (1 μM) and PARP-1 F1 + F2 (0–6.25 μM) were carried out using a buffer that contained either 0 mM (blue circles) or 200 mM sodium chloride (red circles). Stochiometric points were deduced as indicated. (c–e) To determine single-strand nick-specific and nonspecific DNA binding affinities for PARP-1 F1 + F2, we carried out fluorescence anisotropy titrations at a DNA concentration of 10 nM. The following DNA ligands were used: (c) 44 nt 5′-phosphorylated fluorescently labeled nicked DNA dumbbell; (d) 44 nt 5′-phosphorylated fluorescently labeled nicked DNA dumbbell with symmetric stem structures; (e) same ligand as in (d) but ligated to form a circular DNA (see Supplementary Table 2 for DNA sequences). Experiments were carried out at increasing ionic strengths (0, 25, 50, 75, 100, 150 and 200 mM sodium chloride), binding data are colored stepwise from blue to red and data fits are shown as continuous lines. (f) Fitted *K*_D_ dissociation constants for single-strand nick-specific (*K*_D1_) and nonspecific binding (*K*_D_ and *K*_D2_) are plotted as a function of ionic strength (numerical values for the fitted dissociation constants are given in Supplementary Table 1). Data were analyzed using either a one-binding-site model or a two-binding-site model as appropriate.^45^ Binding to symmetrically ligated DNA was not detected at 150 and 200 mM NaCl (e) and was therefore only analyzed at sodium chloride concentrations up to 100 mM.

We used a fluorescently tagged version of this ligand (Fig. 1b) to assess the specificity and stoichiometry of the interaction of PARP-1 F1 + F2 with ssDNA breaks. Sedimentation velocity analytical ultracentrifugation (SV AUC) was carried out either for the DNA alone or following addition of a fivefold excess of PARP-1 F1 + F2. The fluorescence detection system used^44^ allowed the DNA and its complexes to be detected selectively, regardless of any excess of unbound protein; Fig. 4a shows the diffusion-deconvoluted sedimentation coefficient distributions obtained by direct fitting of the Lamm equation (see Supplementary Fig. 2). Although the protein was present in fivefold excess, the observed shift upon DNA binding corresponded closely with that expected for a monomeric complex in a 1:1 stoichiometry. In a control experiment, the single-stranded nick was ligated by reaction with T4 DNA ligase to give a circularized DNA molecule, and then binding to PARP-1 F1 + F2 was again tested by SV-AUC under identical conditions. As shown in Fig. 4a, abolition of the nick in this way caused a very significant reduction in binding of PARP-1 F1 + F2, and abolition of the 1:1 complex, although some residual interaction was still evident. Overall, therefore, these data demonstrate clearly that there is specific recognition of ssDNA nicks by monomeric PARP-1 F1 + F2.

To quantify the binding of PARP-1 F1 + F2 to the nicked DNA dumbbell ligand, we next used fluorescence anisotropy measurements. Solutions of protein were titrated into solutions of the fluorescently tagged DNA ligand, and experiments were repeated as a function of ionic strength. Initial experiments employed DNA concentrations (1 μM) higher than the expected *K*_D_ for the protein–DNA interaction so as to characterize stoichiometry. At relatively high ionic strength (200 mM NaCl), these curves were consistent with a single-site interaction leading to a 1:1 complex, whereas at lower ionic strength (0 mM NaCl), under which conditions predominantly electrostatic protein–DNA interactions would be expected to be stronger, a two-phase curve was observed, suggesting that initial formation of a tight 1:1 complex was followed at higher protein ratios by weaker binding to secondary sites leading to a 1:3 complex (Fig. 4b). To quantify the binding affinities for these interactions, the titrations were repeated at much lower DNA concentrations (10 nM; Fig. 4c–e). For the nicked dumbbell DNA, fitting of the resulting binding curves to a two-site model showed a clear separation into high- and low-affinity sites, with the tighter *K*_D_ taking values between 5.7 nM at 0 mM NaCl and 45 nM at 200 mM NaCl, while the weaker *K*_D_ varied from 0.20 μM at low ionic strength to 2.6 μM at 100 mM NaCl (above 100 mM NaCl the weaker binding component was too weak to quantify). As a control, similar experiments were carried out for the ligated (circularized) DNA ligand, and, as expected, these showed that the tighter binding site had been eliminated, thereby demonstrating unequivocally that this corresponds to protein binding at the single-stranded nick. The best quantification of the strength of the weaker protein–DNA interaction was obtained with a version of the ligated DNA dumbbell in which the DNA sequence (and fluorescent tagging) was changed to make the molecule internally symmetric (Fig. 4d–f), thereby allowing a single-site binding model to be used when fitting the binding curves. This analysis yielded a *K*_D_ that varied from 0.12 μM at 0 mM NaCl to 1.1 μM at 100 mM NaCl, corresponding to the weaker component of binding detected in the experiments for the nicked ligand. Such a residual interaction of PARP-1 F1 + F2 with undamaged DNA is not surprising, as PARP-1 is promiscuous in its roles in DNA repair as well as in other processes such as chromatin remodeling. For instance, it has been reported that PARP-1 F1 + F2 interacts with DNA loop structures^16^ as well as with double-stranded DNA in the case of chromatin binding.^46^ However, our SV-AUC and fluorescence anisotropy data demonstrate unequivocally that PARP-1 F1 + F2 recognizes DNA single-strand nicks specifically, with a high affinity and with 1:1 stoichiometry, and establish the complex with the DNA dumbbell ligand as being suitable for structural studies.

### PARP-1 F1 + F2 recognizes different types of DNA damage in a highly similar fashion

We next investigated the binding of PARP-1 F1 + F2 to different types of DNA single-strand breaks using NMR spectroscopy and gel EMSAs. The ligands used (Fig. 1b) were all dumbbell structures, chosen to mimic possible DNA damage states and intermediates occurring during BER or SSB DNA repair pathways; in addition to the 44-nt nicked dumbbell previously described, we used a 45-nt gapped dumbbell carrying either a 5′ or a 3′ phosphate group or a 5′ ribose.

**Fig. 5:**
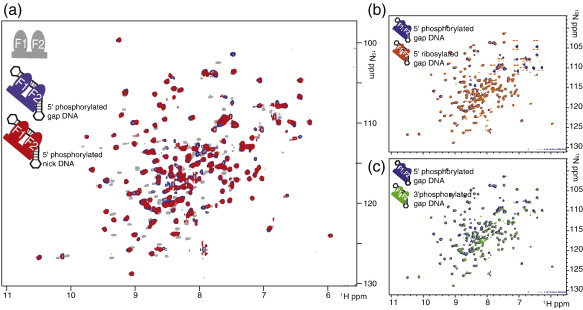
Interaction of PARP-1 F1 + F2 with different types of single-strand breaks. (a) Overlay of [^15^N–^1^H] HSQC spectra of PARP-1 fragment F1 + F2 either in the free state (gray peaks), bound to a 45-nt 5′-phosphorylated gapped DNA dumbbell (blue peaks) or to a 44-nt 5′-phosphorylated nicked DNA dumbbell (red peaks). (b and c) Blue peaks belong to the same spectrum as in (a) overlaid with [^15^N–^1^H] TROSY spectra of PARP-1 fragment F1 + F2 (70% deuterated) bound to either a 45-nt 5′ ribosylated gapped DNA dumbbell (orange peaks) or a 45-nt 3′-phosphorylated gapped DNA dumbbell (green peaks). Closely corresponding peak positions of the DNA-bound proteins strongly indicate that PARP-1 F1 + F2 recognizes all these three types of DNA single-strand breaks in a very similar manner.

Capitalizing on the results of the SV-AUC and fluorescence anisotropy experiments, we were able to reconstitute 1:1 complexes of ^15^N-labeled PARP-1 F1 + F2 and dumbbell DNA ligands suitable for NMR studies. Figure 5a shows a comparison of the HSQC spectra obtained for ^15^N-labeled PARP-1 F1 + F2 in the free state and bound either to the 44-nt nicked dumbbell DNA ligand or to the 45-nt gapped DNA ligand (both having a 5′ phosphate at the strand break). The signals for the two complexes are broader than those for the free protein, consistent with the higher molecular masses (approximately 38 kDa *versus* approximately 24 kDa), but all the spectra are well-resolved and show approximately the number of signals expected for a single monodisperse species; very similar results were obtained for the gapped ligand bearing a 3′ phosphate or 5′ ribose (Fig. 5b and c). It is striking that the patterns of perturbations observed on addition of any of these DNA ligands were almost identical. Mapping of the perturbations onto the structures of the PARP-1 fingers is considered in a following section; for now, the pattern is taken purely as a fingerprint to indicate whether interactions are similar. Chemical shift perturbation data, particularly those for protein amide group chemical shifts, are extremely sensitive to even minor differences in structure or interaction surfaces, so the remarkable degree of correspondence between these patterns must indicate that the mode of interaction of PARP-1 F1 + F2 with all of these ligands is highly similar. Furthermore, it suggests that those parts of the ligands that differ in covalent structure (i.e., those in the immediate vicinity of the single-strand break itself) may not be in direct contact with the protein surface when in the bound state, as otherwise these various shift perturbation patterns would not all be so similar.

Another important feature of these HSQC data is that for all of the DNA ligands tested, the bound-state spectrum of F1 + F2 contains only a single set of backbone amide resonances corresponding approximately to the number of nonproline residues in the molecule. Also, titration of the gapped dumbbell DNA with either F1 + F2 or F2 shows the system to be in intermediate to slow exchange on the NMR chemical shift time scale with respect to exchange between the free and the bound states (see Supplementary Fig. 3). Together, these observations imply that the protein very probably recognizes the directionality of the DNA chain and binds to the damage site in only one orientation, or at least that any minor species bound in the opposite direction is present at a relative concentration below the detection limit of these experiments (approximately 5%). This is perhaps a surprising result, particularly given that binding of PARP-1 to damaged DNA is largely sequence-independent and that the exposed damage features of the DNA ligands may not be in direct contact with the protein (see above). However, given that the system is in slow exchange, the only alternative interpretation for the observation of just one set of bound-state protein signals would be that both orientations are present but each produces identical chemical shift perturbations for all signals of the protein chain. Given the differences between the DNA sequences on the 3′ and 5′ sides of the gap, this seems highly unlikely. Even if these DNA sequence differences do not substantially influence the energetics of protein binding, it seems most unlikely that the different ring current shifts and sequence-dependent conformational variations that the protein would experience in the two different binding orientations would not cause any detectable differences in the chemical shift perturbations observed in the two cases; indeed, preliminary data (not shown) from NMR experiments with other related dumbbell ligands support this view.

**Fig. 6:**
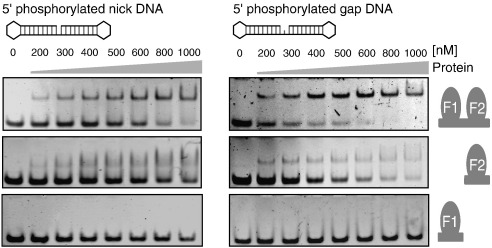
Gel electromobility shift assays. Electromobility gel shift assays of 44-nt 5′-phosphorylated nicked DNA dumbbell (left panel) and 45-nt 5′-phosphorylated gapped DNA dumbbell (right panel) binding to PARP-1 fragments. F1 + F2 binds the most strongly, while F2 binds slightly less strongly, and binding of F1 is not detected in these experiments. Binding to the gapped ligand is consistently slightly stronger than to the nicked ligand. EMSA experiments were performed using 400 nM DNA and increasing protein concentrations (see labeling) of PARP-1 F1 + F2, F2 and F1 (for each binding reaction a volume of 10 μl was loaded). The gel shift experiments allowed a comparison between the different complexes, but binding constants are probably underestimated in comparison to those seen in fluorescence anisotropy measurements due to the nonequilibrium nature of the experiment.

We used gel electromobility shift measurements to compare the strength of DNA binding to PARP-1 F1 + F2 for the cases of the nicked and gapped dumbbell ligands (each with 5′ phosphate). Previous literature suggests that binding of PARP-like zinc fingers to damaged DNA induces a sharp kink at the damage site.^47,48^ It might therefore be expected that the nicked ligand would bind somewhat less tightly than the gapped ligand, since inducing a kink would probably require a greater degree of disruption of base stacking across the damage site for the nicked ligand than for the gapped ligand. Our results confirmed this expectation. The gel shifts shown in Fig. 6 demonstrate that the nicked ligand binds approximately twofold less tightly to PARP-1 F1 + F2 than does the gapped ligand; for this reason, the gapped ligand was used in all subsequent NMR experiments.

### Recognition of DNA single-strand breaks by PARP-1 occurs predominantly through F2

To address the question of the differential roles of PARP-1 F1 and F2 in DNA damage recognition, we compared the binding of the separated finger domains F1 and F2 to that of the two-finger fragment F1 + F2. It quickly became clear that the majority of the DNA-binding affinity of the F1 + F2 fragment resides in F2. Figure 6 shows gel shifts measured for all three protein fragments, both with the nicked and the gapped ligands, and while F2 shows shifts consistent with homogenous complex formation comparable to that by PARP F1 + F2, the results for F1 show that it barely binds at all under the conditions of the gel shift experiments. Corresponding results were also obtained in fluorescence anisotropy measurements. In initial experiments, we widened the comparison to include also the single zinc finger of DL3, the behaviour of which was found to be more similar to that of PARP-1 F2 than of PARP-1 F1 (see Supplementary Fig. 4a). Since binding measured by gel shift experiments depends not only on the equilibrium constant but also the off-rate for the complex, which can lead to an underestimation of dissociation constants (see Fig. 6), we quantified the interaction of PARP-1 F1 and F2 with the DNA dumbbell using fluorescence anisotropy measurements. F2 binding was measured at differing ionic strengths and applied the same two-site binding model to analyze the results as we had used for the data from the F1 + F2 fragment. Although the fitting was more difficult in this case, there was still evidence for tight and weak binding components at low and intermediate ionic strengths, the tighter component varying from 0.13 μM at 50 mM NaCl to 0.15 μM at 100 mM NaCl, and the weaker from 1.7 μM at 50 mM NaCl to 10.6 μM at 100 mM NaCl. This shows that F2 binds the nicked DNA ligand with approximately 10-fold lower affinity than does F1 + F2, so at higher ionic strengths (and at the total DNA concentration used in these experiments, i.e., 10 nM), overall binding of F2 was too weak to be analyzed in this way (Supplementary Fig. 4b and c). However, F1 alone binds the DNA with an even weaker affinity as quantified by fluorescence anisotropy measurements (at least 100-fold weaker than for F1 + F2; see Supplementary Fig. 4d).

**Fig. 7:**
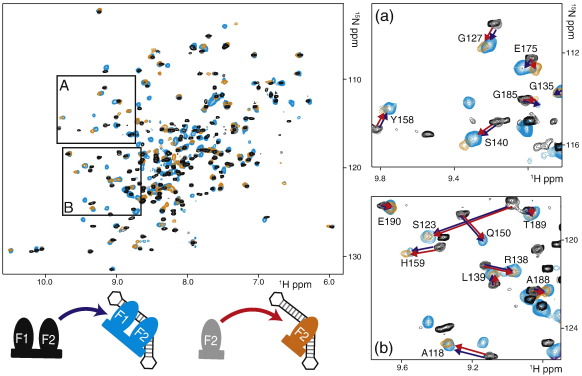
Comparison of F2 and F1 + F2 in interaction with DNA single-strand breaks. Overlay of [^15^N–^1^H] HSQC spectra of PARP-1 fragment F1 + F2 and F2 either in the free state (F2, gray peaks; F1 + F2, black peaks) or in the DNA-bound state (F2, orange peaks; F1 + F2, blue peaks). Two expansions are shown in (a) and (b). The two 1:1 protein–DNA complexes (100 μM) were each reconstituted under the same experimental conditions using the 44-nt 5′-phosphorylated DNA dumbbell with a single-stranded nick. DNA binding caused essentially identical chemical shift perturbations for F2 alone (red arrows) as it did for F2 in the context of a two-zinc-finger construct (F1 + F2; blue arrows), as exemplified in the expansions. These data demonstrate that PARP-1 F2 retains the same DNA binding configuration regardless of the presence or absence of F1.

**Fig. 8:**
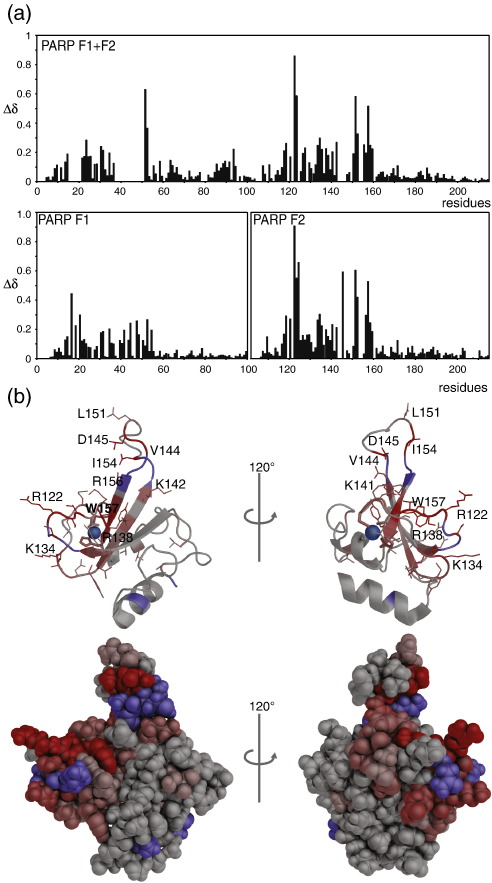
Chemical shift perturbation analysis. (a) Histograms of backbone amide chemical shift perturbations for F1 + F2 (upper panel), F1 and F2 (both lower panels) upon binding to the 45-nt 5′-phosphorylated gapped DNA dumbbell. Perturbations are calculated as Δδ = √[(δ^1^H)^2^ + (δ^15^N ÷ 5)^2^] (b) Chemical shift perturbations from (a) mapped onto the solution structure of F2 in both cartoon and space-filling representations. Perturbations were divided into five categories according to the SD over all perturbations: unaffected (0–0.5 × SD), weak (0.5–1 × SD), medium (1–1.5 × SD), strong (1.5–2 × SD) and very strong (> 2 × SD) and colored accordingly from gray (unaffected) to red (very strong). On the cartoon view, side chains of perturbed residues are drawn as thin lines. Residues for which no chemical shift perturbation could be determined due to missing assignments are colored blue.

Consequently, we asked next whether PARP-1 F2 in isolation binds DNA single-strand breaks in the same configuration as it does when in the context of the F1 + F2 fragment. We used the same conditions as for the F1 + F2 fragment to reconstitute a 1:1 complex of ^15^N-labeled PARP-1 F2 with the gapped dumbbell DNA ligand. The resulting HSQC spectrum was well resolved and showed approximately the number of signals expected for a single monodisperse species. Furthermore, as shown in Fig. 7, the perturbations of signals arising for F2 upon binding to the gapped DNA ligand are essentially identical, regardless of whether the protein fragment used in the titration includes F1 or not. Insets A and B in Fig. 7 show this very close correspondence for several specific cases that are particularly clear because they occur in spectral regions that are free from overlap, but a more detailed analysis shows that a similar correspondence holds for all of the residues of F2 where the comparison can be made (see Fig. 8a). As in the case of the comparison of different DNA ligands, it is noteworthy that this very close correspondence implies that the binding interfaces must be extremely similar in the two cases, since chemical shift is a very sensitive reporter of even minor local differences in structure. These data show very clearly that the manner in which F2 recognizes the DNA damage site is essentially identical in fragments F2 and F1 + F2.

It is also significant that a similar comparison made for F1 gives a quite different outcome. For isolated F1, spectra recorded under the same conditions as used for fragments F1 + F2 and F2 showed severe line-broadening characteristic of an intermediate-rate exchange process, in itself an indication of weaker binding (see Supplementary Figure 5). Under conditions of higher ionic strength where binding is weaker still, relatively well-resolved spectra were obtained and signal perturbations could be determined. Because the NMR titration experiments are necessarily carried out at high protein concentrations, appreciable signal perturbations are expected even for relatively weak interactions, so it is not surprising that perturbations are visible for F1 even under these conditions (Fig. 8). However, they are systematically smaller than those seen in the corresponding experiment with F2, probably reflecting at least in part the lower extent of saturation for binding of F1. Significantly, some of the DNA-induced perturbations seen for F1 differ according to whether it is present alone, in the context of fragment F1, or linked to F2, in the context of fragment F1 + F2 (Fig. 8a). Interpretation of this result is not straightforward, as the ionic strengths are not identical for experiments with F1 as for those with F1 + F2 and the binding site(s) for F1 are not characterized in any of these experiments; indeed we have no indication from any of this work that F1 necessarily forms a homogeneous complex with the DNA. This is in clear contrast with the results for F2 and underscores the conclusion that it is F2 that wins the competition for binding to the DNA strand-break when the DNA ligand is titrated against the F1 + F2 protein fragment.

### Chemical shift perturbations highlight the nature of the interactions with DNA

To determine the chemical shift perturbations upon DNA binding, it necessary to obtain assignments for the signals of the PARP-1 F1, F2 and F1 + F2 fragments in the bound state, which was done using a set of experiments similar to that employed for the free fragments described earlier. However, the broader lines for these complexes necessitated use of partially deuterated protein (approximately 70% ^2^H) to reduce dipolar relaxation rates and thereby allow the various transverse relaxation-optimized spectroscopy (TROSY)-based triple-resonance experiments used for assignment to succeed. An essentially complete assignment of F1 + F2 was obtained, except for parts of F1 (notably parts of strands β2, β3, L2 and loop L1). As described above, signals observed for the complexes with either the 44-nt nicked or the 45-nt gapped ligands were extremely similar to one another, and assignment experiments were all carried out using the gapped ligand. The chemical shift perturbations for the F1, F2 and F1 + F2 fragments upon addition of the gapped ligand are shown in Fig. 8a, and are mapped onto the structure in Fig. 8b.

For F2, there are a large number of residues showing perturbations, but essentially all of them are located in the first half of the sequence, within the region of the triple stranded β-sheet and the loops connecting its strands. The largest of these perturbations occur in strand β3, loop L2 and that part of the long loop L1 that is closest in space to strand β3, suggesting that these elements, which are adjacent on the surface of the structure of F2, may form the primary binding interface for DNA damage recognition. The residues involved are mainly basics (e.g., Arg122 and Lys134 in loop L1 and Arg138, Lys142 and Arg156 in the sheet) and some hydrophobics (e.g., Leu151 in loop L2 and Ile154, Trp157 and Tyr158 in the sheet). In addition, NH signals for hydrophobic residues near the ends of loop L2 (specifically, Met143–Val144 and Met153–Ile154) remain unassigned in the bound state due to severe exchange broadening, but perturbations of their methyl signals show clearly that at least Val144 and Ile154 are also involved in DNA binding (data not shown). Overall, this mapping affords a much more detailed view of the DNA-binding interface than was seen previously for the related case of the single finger from DL3,^31^ for which only a more limited set of bound-state assignments were available. For instance, the present data show clearly the involvement of loop L1, not detected for the case of DL3. However, it is likely that such apparent differences reflect the different extents of assignments for the bound state rather than any intrinsic difference in the DNA-binding surfaces in the two proteins.

Residues within the two helices and loop 4 of F2 show only insignificant perturbations upon addition of DNA, strongly suggesting that these regions of the structure do not participate in DNA binding. This is entirely consistent with the results obtained previously for DL3.^31^

As described earlier, the pattern of perturbations seen for the signals of residues of F2 are essentially identical, regardless of whether F2 is present alone or in the context of the two-finger fragment F1 + F2. The same is not true for all signals of residues in F1, some of which show different perturbations upon DNA interaction in the case of isolated F1 from those seen for the case of the two-finger fragment F1 + F2. Interpretation of these results for F1 is difficult, but it might suggest that the mode of interaction of F1 is somewhat different when it is tethered to F2 from that when it is not. Nonetheless, it is clear at an overall level that essentially the same regions of the structure of F1 are involved in DNA interactions as for F2, and it is noticeable that the residues of F1 that suffer severe line broadening upon DNA interaction in the context of the F1 + F2 fragment are in the same regions as those arising from the parts of the structure that interact the most strongly in the case of F2. Similarly, it is clear that for each of the fingers in isolation, the regions unaffected by DNA interaction (i.e., the two helices and loop L4) are again common between F1 and F2. Intriguingly, however, some residues of helix 2 of F1 show perturbations only when present in the two-finger fragment F1 + F2. In the absence of more detailed structural information it is very difficult to interpret this result, but it is tempting to speculate that this might indicate a DNA-dependent interfinger contact. This is far from being the only possible interpretation, however.

### Differences between PARP-1 fingers 1 and 2: Mutational study

To help validate the proposed DNA-binding interface and rationalize the pronounced differences observed between the DNA-binding properties of F1 and F2, we carried out a study of mutants of PARP-1 F2.

**Fig. 9:**
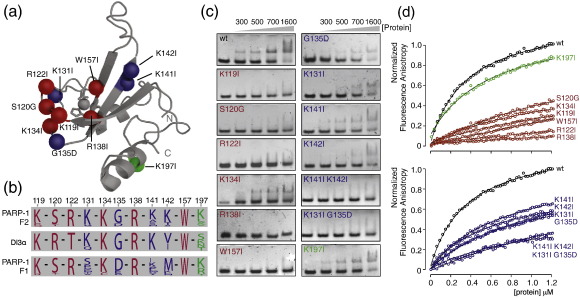
Mutational analysis of PARP-1 F1 and F2. Two groups of mutations were selected. The first group (shown in red) includes residues that are similarly conserved between PARP-1 F1, PARP-1 F2 and the closely related zinc finger of DL3; mutations of these residues abolishes DNA-binding in every case (except K134I where it is significantly reduced). The second group (shown in blue) includes residues that are similarly conserved in PARP-1 F2 and DL3, but different in PARP-1 F1; single mutations in this group reduce DNA-binding affinity, while selected double mutations abolish it. As a control (shown in green), we mutated Lys197, which is not part of the DNA binding surface (see Fig. 8). (a) Mutations shown on the solution structure of PARP-1 F2. (b) Sequence logos produced using the multiple alignment of PARP-1 F1, PARP-1 F2 and DL3 given in Ref. 31. Residues Arg122 and Ser120 (F2 numbering) are conserved in PARP-1 F1 and F2 but transposed in DL3; however, they could still play corresponding roles in all three fingers. (c and d) DNA binding of wild-type PARP-1 F2 and mutants was compared using electrophoretic mobility gel shifts (c) and fluorescence anisotropy titrations (d). The gel experiments used the 45-nt 5′-phosphorylated DNA dumbbell ligand, and the fluorescence anisotropy titrations used fluorescently labeled 44-nt 5′-phosphorylated DNA dumbbell ligand (25 nM) in a buffer containing 50 mM Tris (pH 7), 25 mM NaCl, 150 μM ZnSO_4_ and 4 mM DTT. Fluorescence anisotropy data were analyzed using a one-binding-site model and the resulting apparent dissociation constants are given in Supplementary Table 2.

In a first group of mutations, we selected surface residues of F2 that are highly conserved amongst PARP-like zinc fingers (based on the alignment in Ref. 31) and are involved in the DNA interaction, as detected by the NMR chemical shift perturbation experiments. These comprised Lys119, Ser120, Arg122 and Lys134 located in the long loop 1, and Arg138 and Trp157 on the surface of the β-sheet. We chose isoleucine as the replacement residue in most of these cases, since (a) we wanted primarily to test the importance of the presence of the basic moiety of particular lysine or arginine side chains and therefore wished not to perturb the nearby structure by simultaneously removing the hydrophobic parts of those side chains, and (b) isoleucine was also the replacement residue used in a few key cases in some earlier work on PARP-1 mutants.^49^ The folding and structural similarity of each mutant was checked using ^15^N HSQC experiments, which showed that in all cases the structure of the protein is preserved (although, not unexpectedly, mutations in the sheet cause stronger chemical shift perturbations than those in the loop; Supplementary Fig. 6). All of these mutations caused a significant reduction in DNA binding by F2 (except for Lys134Ile, which caused a less severe reduction), as assessed by gel electromobility shift assays and fluorescence anisotropy measurements (Fig. 9; apparent dissociation constants are given in Supplementary Table 2). This confirms the importance of the triple-stranded β-sheet and the long loop L1 in recognition of DNA single-strand breaks. As a negative control, we also mutated Lys197, since this residue is located on the opposite side of the molecule from the DNA binding surface; as expected, this mutant showed an essentially identical DNA-binding affinity to that of the wild-type protein (Fig. 9). Our results for the Arg138Ile mutation are consistent with those of a previous study in which the same mutation was introduced into a larger PARP-1 fragment (residues 1–373), where it also abolished DNA binding, suggesting that neither F1 nor F3 of PARP-1 can compensate for the function of F2.

In a second group of mutations, we addressed the question of the origin of the difference in DNA-binding affinity between F2 and F1. Given that F2 displays DNA binding characteristics comparable to those of the single zinc finger of DL3, we reasoned that it would be interesting to look for ways in which these two fingers resembled one another but differed consistently from F1 of PARP-1. Comparing the sequences, one can indeed identify surface residues in the DNA-binding regions that are well-conserved across species in all three fingers, but which are of a markedly different type in PARP-1 F1 from that found in both PARP-1 F2 and DL3 (Fig. 9b). These include Lys141 and Lys142, which form a basic patch at the end of strand 2, and Lys131 and Gly135, which are located in the long loop L1. Gly135 is highly conserved in PARP-1 F2 and DL3, whereas PARP-1 F1 contains a conserved aspartate at the equivalent position, prompting us to choose aspartate as the replacement residue for this mutation. Interestingly, mutation of any of the residues in this second group caused reduction in DNA binding to an intermediate extent. We speculated, therefore, that the difference in binding affinity between F1 and the other two fingers was probably due to combinatorial effects of these residues. We therefore made and tested two double mutants, Lys141Ile/Lys142Ile and Lys131Ile/Gly135Asp, and for both of these we found that binding to the dumbbell DNA was abolished under the test conditions. Both of these double mutants change the local charge by two units, suggesting that the tighter DNA strand-break binding by F2 and DL3 is most probably due at least in part to local differences in their electrostatic surfaces relative to that of F1. This interpretation is reinforced by examination of the calculated electrostatic surface potentials shown in Supplementary Figure 7.

## Discussion

Although it is nearly 50 years since PARP-1 was discovered, the mechanisms by which it recognizes damaged DNA and subsequently becomes activated remain largely uncharacterized. Here, we investigate how the two N-terminal zinc fingers of PARP-1, F1 and F2, interact with DNA single-strand breaks. We show that recognition of such breaks is predominantly accomplished by the second of the two zinc fingers and that binding by either the F2 or the F1 + F2 fragments of PARP-1 occurs as a monomer. We show that recognition by F2 occurs in an essentially identical fashion regardless of the presence or absence of F1 and also that a variety of DNA ligands harboring different types of single-strand breaks are equivalently recognized.

It is likely that the recognition events described here between the F2 or the F1 + F2 fragments of PARP-1 and our dumbbell DNA ligands *in vitro* correspond closely to the first steps during the process of activation of full-length PARP-1 by DNA single-strand breaks *in vivo*. The DNAase-1 footprint of full-length PARP-1 binding to a single-strand break is known to be identical to that of the F1 + F2 fragment (± 7 nt around the break)^25^ and is fully accommodated by our DNA dumbbell ligands. Consistent with this, it has been found that *in vivo* the F1 + F2 fragment contains all of the DNA-damage binding activity relevant to activation, since activation of full-length PARP-1 is suppressed due to competitive binding when an excess of the F1 + F2 fragment is present, either as a result of cleavage of full-length PARP-1 by caspase-3 and -7 during apoptosis or due to overexpression of the fragment.^28,29^ We find that F1 + F2 binds DNA single-strand breaks *in vitro* with dissociation constants in the nanomolar range, while PARP-1 is a highly abundant nuclear protein for which a micromolar concentration has been estimated in the nucleus of eukaryotic cells (approximately 2 × 10^5^ PARP-1 molecules per nucleus^50^), entirely consistent with its proposed role as an efficient sensor of DNA single-strand breaks. Intriguingly, this function is relevant in both the BER and the SSBR DNA repair pathways^13,51^ as well as in transcriptional control,^52^ implying that PARP-1 must recognize DNA single-strand breaks that have quite different chemical structures. Indeed, a recent study has shown that PARP-1 recognizes even abasic sites and is activated upon subsequent strand incision by APE1.^53^ Our data suggest that despite the different functional groups present at these different types of strand breaks, the DNA-binding domain of PARP-1 recognizes them in an essentially similar way, helping to rationalize how it can achieve its function in all these varied cases. Electron microscopy studies have shown that on binding to intact PARP-1 a very sharp kink is induced in the DNA,^47^ suggesting that what PARP-1 recognizes may be the ability of damaged DNA to form a tighter bend than can be accommodated by an intact double helix.

The tighter binding of DNA single-strand breaks by F2 than by F1 raises the question of the differential functional roles of the two fingers in full-length PARP-1. Our chemical shift perturbation data showed clearly that the nature of DNA-recognition by F2 is largely unaffected by the presence or absence of F1, and it has previously been shown that F1 is dispensable for DNA binding, whereas F2 is not. For instance, Gradwohl *et al.* showed for the F1 + F2 fragment that disruption of the structure of F2 by mutation of zinc-binding residues greatly reduced binding to DNA single-strand breaks, whereas similar mutations in F1 did not,^32^ and similar results were obtained for a construct of PARP-1 that contains all three zinc fingers.^49^ Moreover, DL3 contains only one PARP-like zinc finger, which intriguingly leaves a DNase I footprint around single-strand breaks similar to that of PARP-1,^54^ suggesting that a single finger is sufficient for DNA single-strand breakage recognition. This parallel between the DL3 finger and PARP-1 F2 is reinforced by the similarity we see in the patterns of chemical shift perturbations upon binding of either to DNA single-strand breaks. Putting all of this together, we conclude that DNA single-strand break recognition of PARP-1 is primarily achieved by F2.

The picture concerning activation is much less clear-cut. For instance, Ikejima *et al*.^34^ showed that ablation of F1 by mutation of metal-binding cysteines in full-length PARP-1 eliminated activation by either DNA single-strand or double-strand breaks, whereas ablation of F2 eliminated only activation by single-strand breaks. Similarly, a recent paper by Altmeyer *et al*.^55^ shows through deletion analysis that F1, but not F2, is essential for activation. Overall, it seems likely both from our results and from previous work that at least for interaction of PARP-1 with DNA single-strand breaks, the principal functional role of F1 is to play a part in transmitting the signal for activation of PARP-1's catalytic activity subsequent to DNA single-strand breakage recognition by F2.

It remains to be determined how F1 achieves this role in cooperation with F2 and in context of the full-length PARP-1. Our results suggest that an isolated F1 fragment probably binds to our DNA dumbbell ligands in a mode qualitatively similar to that seen for F2 binding, although with much lower affinity, and we also show that F1 does make some contribution to the DNA-binding affinity of the F1 + F2 fragment. However, in the context of the F1 + F2 fragment, F1 must interact with a part of the ligand that is not already occupied by F2.

Since activation of PARP-1 by damaged DNA or repair intermediates *in vitro* does not require additional factors, it must presumably involve some DNA-dependent change in interdomain interactions within PARP-1 itself, either within a single molecule or in an oligomeric assembly. While the overall nature of these interactions remains to be determined, some isolated details are starting to emerge. For instance, inactivating point mutations in F3 have been reported by Trucco *et al.*^33^ and, more recently, by Langelier *et al*.,^36^ while Altmeyer *et al.*^55^ showed by domain deletion that not only F3 but also the WGR domain is essential for activation. Our results show that the chain of interdomain interactions involved in activation is most likely to start with F2, which is fully consistent with recent results of Lilyestrom *et al.*,^56^ who showed, using low-angle X-ray scattering experiments, that domains F1, F2 and F3 probably acquire additional mutual interactions upon DNA binding. Our results may help shed light on this process; highly conserved surface residues of F2 that are remote from the DNA-binding region are prime candidates to be involved in interdomain interactions with either F1 or F3 in the DNA-bound state. Although to our knowledge there are no data on the role of such residues in activation, it is interesting that at least one corresponding position in F1 is involved. Trucco *et al.*^33^ showed that mutation of Leu77 inactivates PARP-1, and although this residue is buried, its immediate neighbour Trp79 is solvent-exposed and a good candidate for interdomain interactions.

Others have pointed out^36,56^ that the two conformations observed for the C-terminal part of F3 (interacting in trans with a partner chain in the crystallographic dimer^23^ or with its own chain in cis in the NMR structure^24^) might have a functional significance; mutation of residues in the dimer interface lead not to inactivation of PARP-1's catalytic domain, but rather to suppression of PARP-1's chromatin compaction activity.^36^ This might suggest that this conformational change might act as a functional switch with a double function in controlling PARP-1's activity in transcriptional regulation, whereby in the “on” state, modification with PAR leads to decondensation of chromatin structure during transcription, whereas in the “off” state, not only is PAR synthesis absent, but also chromatin is compacted to further suppress transcription.

Another key aspect of our results is that binding of either the F2 or the F1 + F2 PARP-1 fragments to these DNA dumbbell ligands occurs as a monomer. There have been several suggestions previously that activation of PARP-1 involves dimerization, but it is important in this context to distinguish between the DNA-binding interaction itself and activation. The original evidence for a possible role for dimerization during activation came from the bimolecular kinetics observed for the automodification reaction,^35^ which in turn has been taken to suggest that PARP-1 assembles to form a dimer upon binding to a DNA-damage site. However, such assembly and subsequent activation of PARP-1 would not necessarily have to be mediated through dimerization of the DNA-binding domain itself. Indeed, PARP-1 can be activated as a heterodimer, for instance, in complex with PARP-2 that lacks the whole N-terminal zinc-finger region and BRCT domain,^57^ and our results are consistent with those of Lilyestrom *et al.*, who show clearly that binding of a PARP-1 fragment comprising the F1, F2, F3 and BRCT domains to various DNA damage sites occurs as a monomer.^56^ In contrast, Pion *et al.*^37^ have reported dimerization of the F1 + F2 fragment at a 3′ overhang (called a 5′ recessed end in their paper), and their work has often been cited in support of the proposal that DNA-mediated dimerization of PARP-1 occurs. It is not straightforward to reconcile their results with those of ourselves and of Lilyestrom *et al*., but one possible explanation could be that binding of PARP-1 to different DNA ligands results in different binding stoichiometries. Thus, it appears that the process of activation of PARP-1 catalytic domain may not involve dimerization of the DNA-binding domain.

In conclusion, it is clear that a full description of the activation mechanism of PARP-1 will require significant further work to characterize its interdomain interactions and their DNA dependence, both at a structural and at a functional level. Nonetheless, the results presented here provide valuable insights into recognition of chromosomal DNA single-strand breaks by the PARP-1 DNA-binding domain, the crucial first step of the activation process.

## Materials and Methods

### Expression and purification of PARP-1 zinc finger constructs

DNA coding for different fragments of human PARP-1 (F1, residues 1–108, F2, residues 103–21 and F1 + F2, residues 1–214) was subcloned into a Pet13 vector (for fragment F1 + F2) or a Pet28a vector (for fragments F1 and F2) using BamHI and NcoI restriction sites. Proteins were recombinantly expressed and enriched with stable isotopes (^15^N, ^13^C and/or ^2^H) using *E. coli* BL21-CodonPlus(DE-3)-RP cells (Stratagene) cultured in M9 minimal medium. Protein purification was carried out as described in Supplementary Materials and Methods.

### Preparation of DNA dumbbell ligands

HPLC purified DNA dumbbell ligands were obtained from the in-house DNA synthesis facility of the MRC Laboratory of Molecular Biology (Cambridge, UK). The sequences are given in Supplementary Table 2. DNA ligands were further purified using denaturing polyacrylamide gel electrophoresis according to the protocol of reference.^58^ Correct refolding of DNA ligands into a monomeric dumbbell conformation was confirmed by NMR spectroscopy, as described in Ref. 31. Ligation of DNA dumbbell ligands was carried out as described in Ref. 59 with adapted experimental conditions (see Supplementary Materials and Methods).

### NMR spectroscopy

All NMR data were acquired on Bruker Avance 800, DMX600 and DRX500 spectrometers, each equipped with a triple-resonance (^1^H/^15^N/^13^C) cryoprobe. All NMR experiments on free PARP-1 fragments were carried out at 27 °C using ^15^N- or ^15^N, ^13^C-labeled protein samples adjusted to 50 mM [^2^H_11_]Tris (pH 7.0), 200 mM NaCl, 150 mM ZnSO_4_, 4 mM [^2^H_6_]DTT and 5% D_2_O (v/v), except that for the RDC measurements the NaCl concentration was increased to 430 mM and filamentous phage Pf1 (ASLA Biotech) was added to a final concentration of 14 mg/ml. Protein concentrations were 0.5–1 mM for NOESY experiments used to derive structural constraints, 0.45 mM for the ^15^N relaxation experiments and 0.15 mM for experiments to measure RDC values. Resonance assignments for protein fragments in the free state were made using a standard suite of triple-resonance experiments. Samples of PARP-1 F2 and F1 + F2 fragments bound to DNA ligands were reconstituted in 50 mM [^2^H_11_]Tris (pH 7.0), 150 μM ZnSO_4_, 4 mM [^2^H_6_]DTT and 5% D_2_O (v/v), and their NMR signals at 37 °C were assigned as described in Supplementary Materials and Methods. For F1 bound to DNA, a similar procedure was followed except that 200 mM NaCl was also present in the buffer. ^1^H, ^15^N and ^13^C chemical shifts were calibrated using sodium 3,3,3-trimethylsilylpropionate (TSP) as an external ^1^H reference. Amide group chemical shift perturbation data for PARP-1 F1, F2 and F1 + F2 were calculated as Δδ = √[(δ^1^H)^2^ + (δ^15^N ÷ 5)^2^].

### Structure determination of PARP-1 F1 and F2

Structures of PARP-1 F1 and F2 were determined with a two-stage protocol essentially as described previously.^60^ Initial structures were calculated with the program ATNOSCANDID,^61^ taking as input the NOESY spectra, resonance assignments, the amino acid sequence and dihedral constraints derived from the program TALOS. The NOE distance constraints from the final round of ATNOSCANDID calculations, together with TALOS-derived dihedral constraints, were used as input to calculations using the program XPLOR-NIH,^62^ employing explicit zinc atoms. In an additional set of calculations, we also used amide group RDC data together with the ISAC refinement protocol of Sass *et al.*^42^ to derive alignment tensors for the RDC analysis to test for interaction between the fingers. For deposition, the XPLOR-NIH structures calculated without using RDC constraints were used as input to a final stage of refinement in the program AMBER, using a full force field and generalized Born solvent representation.^63^ Details of the structure calculation protocols are given in Supplementary Materials and Methods. The program CLUSTERPOSE was used to calculate the mean rmsd of ensembles to their mean structure,^64^ and structures were visualized with the program PyMOL.^65^

### Electromobility shift assays

Band shift assays were carried out with native 7.5% polyacrylamide gels (1.0 mm × 10 well, 10 × 10.5 cm). TB buffer (0.5×) with 5% glycerol was used for gel preparation and as running buffer. Prior to usage, wells were thoroughly flushed with running buffer and gels were prerun at 55 V for 20 min at 4 °C. DNA ligand (400 nM) was mixed with protein in binding buffer [50 mM Tris–HCl (pH 7.4), 150 μM ZnSO_4_, 4 mM DTT, 10% glycerol] in a total volume of 10 μl. Prior to gel electrophoresis, samples were incubated for 30 min at room temperature. Gel electrophoresis was conducted for 50 min with 55 V at 4 °C. DNA was stained with ethidium bromide.

### Fluorescence anisotropy measurements

Fluorescence anisotropy measurements were performed on an LS 55 Luminescence Spectrometer (Perkin Elmer, Ltd.) equipped with a Hamilton Microlab® 500 Series titrator (Hamilton, Inc.) controlled by laboratory software FLUOPE (D. Veprintsev, MRC Center of Protein Engineering, Cambridge, UK) essentially as described in Ref. 66. The excitation and emission wavelengths used were 480 and 530 nm, respectively, and the slit widths for excitation and emission were 15 and 20 nm, respectively. Experiments were performed at 25 °C in 50 mM Tris–HCl (pH 7), 150 μM ZnSO_4_, and 4 mM DTT with an NaCl concentration of 0–200 mM. Protein solutions were titrated in steps of 5 μl into a cuvette (119.004F-QS, Hellma) containing 1000-μl solution of fluorescein-dT-labeled DNA dumbbell ligands (for sequences, see Supplementary Table 2). Depending on the experiment, protein concentrations were 5–20 μM and DNA concentrations were 10 nM–2 μM. Data were treated and analyzed essentially as described in Ref. 66. For data analysis, fluorescence anisotropy values were normalized between 0 for free DNA and 1 for the maximal measured value of the respective protein–DNA complex. The program Kaleidagraph (Synergy) was used to obtain a fit of the fluorescence anisotropy data to either a one-binding-site model, *r*_obs_ = *r*_max_[P]/(*K*_D_ + [P]), or a two-binding-site model, *r*_obs_ = *r*_1max_[P]/(*K*_D1_ + [P]) + *r*_2max_[P]/(*K*_D2_ + [P])^45^ (for further details see Supplementary Material and Methods).

### Analytical ultracentrifugation

The SV-AUC experiments were performed with an Optima XL-I ultracentrifuge with an An50Ti rotor (Beckmann-Coulter), an Aviv fluorescence detection system (Aviv Biomedical) and SedVel60K-FDS fluorescence velocity cells (Spin Analytical) with sample volumes of 65 μl. Experiments were conducted at 20 °C using concentrations of 0.5 μM 44-nt fluorescein-labeled DNA dumbbell and 2.5 μM PARP-1 F1 + F2 in a buffer containing 50 mM Tris (pH 7), 150 mM NaCl, 150 μM ZnSO_4_ and 4 mM DTT. Concentration gradients were measured at a rotor speed of 45,000 rpm every 78 s at an excitation wavelength of 488 nm and a detection of emitted light > 505 nm. Continuous diffusion-deconvoluted sedimentation coefficient distributions *c*(*s*) were obtained by direct fitting of the Lamm equation using the software sedfit.^67^ Data fitting was performed with fixed partial specific volumes for the respective sample and a floating frictional ratio. Partial specific volumes of the DNA–protein complex were calculated as mass-weighted averages of the individual components assuming the partial specific volume of DNA to be 0.54 ml/g.^68^ Buffer density and viscosity as well as the partial specific volume of PARP-1 F1 + F2 were calculated using the program SEDNTERP.^69^

### Mutational analysis of PARP-1 F2

Mutations of the DNA coding for the amino acid changes of PARP-1 F2 listed in Fig. 9 were introduced with a QuikChange II Site-Directed Mutagenesis Kit (Stratagene) according to the manufacturer's recommendations and verified by DNA sequencing. Mutant proteins were expressed and purified as described above, using M9 minimal medium supplemented with ^15^NH_4_Cl (0.5 g/L) (Sigma Aldrich Isotec). Folding of purified ^15^N labeled proteins was verified by NMR spectroscopy using [^15^N–^1^H] HSQC spectra. DNA binding of mutants was tested by electromobility band shift assays using 5′-phosphorylated 45-nt gapped DNA dumbbell ligand as described above.

### Accession numbers

^1^H, ^15^N and ^13^C NMR resonance assignments for PARP-1 1–108 and PARP-1 103–214 at 27 °C and pH 7.0 have been deposited with the BioMagResBank (Madison, WI) with accession numbers 17157 and 17158, respectively. Atomic coordinates for the ensembles of 30 final structures of PARP-1 1–108 and PARP-1 103–214 have been deposited in the Protein Data Bank with accession numbers 2l30 and 2l31, respectively.

#### Note added in proof

While this article was at the proof stage, a paper describing crystal structures of PARP-1 F1 and F2 (separately) bound to DNA blunt ends was published (Langelier *et al*., *J. Biol. Chem.*, doi:10.1074/jbc.M110.202507) with related pdb entries 3od8, 3oda, 3odc and 3ode.

## Figures and Tables

**Table 1:** Structural statistics for the deposited ensembles of NMR structures for finger 1 (F1) and finger 2 (F2) of human PARP-1

	F1	F2
*Structural restraints*		
NOE-derived distance restraints		
Intraresidue	323	298
Sequential	494	523
Medium (2 ≤ |*i* − *j*| ≤ 4)	309	463
Long (|*i* − *j*| > 4)	628	777
Total	1754	2061
Dihedral restraints		
phi	51	56
psi	54	61

*Statistics for accepted structures*		
Number of accepted structures	30	30
Mean AMBER energy terms (kcal mol^− 1^ ± SD)		
*E*(total)	− 3737.1 ± 9.7	− 4596.8 ± 12.1
*E*(van der Waals)	− 790.8 ± 11.7	− 815.7 ± 9.7
*E*(distance restraints)	20.0 ± 1.4	18.1 ± 1.7
*E*(dihedral restraints)	1.3 ± 0.5	5.3 ± 1.1
Distance restraint violations > 0.2 Å (average number per structure)	2.9 ± 1.0	2.0 ± 1.0
Angle restraint violations > 5° (average number per structure)	1.8 ± 0.8	6.3 ± 1.5
rms deviations from the ideal geometry used within AMBER		
Bond lengths (Å)	0.0101	0.0098
Bond angles (°)	2.19	2.06

*Ramachandran statistics*		
Most favoured (%)	90.4	86.4
Additionally allowed (%)	9.6	13.4
Generously allowed (%)	0.0	0.0
Disallowed (%)	0.0	0.1
		
*Average atomic rms deviations from the average structure (± SD) (Å)*		
	Residues 7–93	Residues 109–200
N, C^α^, C atoms	0.50 ± 0.12	0.54 ± 0.15
All heavy atoms	0.88 ± 0.12	0.92 ± 0.12
	Residues 7–40, 46–93	Residues 109–144, 152–200
N, C^α^, C atoms	0.39 ± 0.07	0.37 ± 0.10
All heavy atoms	0.82 ± 0.10	0.79 ± 0.09
