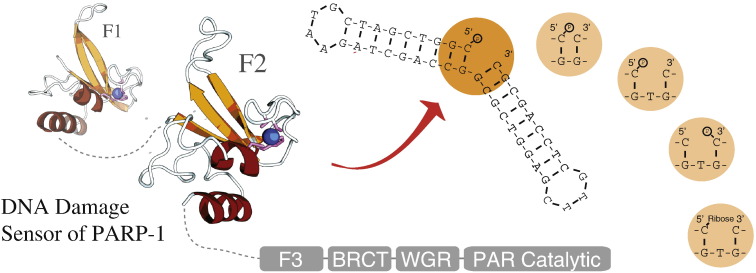# Corrigendum to “The DNA-Binding Domain of Human PARP-1 Interacts with DNA Single-Strand Breaks as a Monomer through Its Second Zinc Finger” [*J. Mol. Biol.* 407 (2011) 149–170]

**DOI:** 10.1016/j.jmb.2012.03.018

**Published:** 2012-06-08

**Authors:** Sebastian Eustermann, Hortense Videler, Ji-Chun Yang, Paul T. Cole, Dominika Gruszka, Dmitry Veprintsev, David Neuhaus

**Affiliations:** MRC Laboratory of Molecular Biology, Hills Road, Cambridge CB2 0QH, UK

The authors regret that there is an typographical error in the DNA sequence shown in Fig. 1b and the graphical abstract of this paper: The G and C bases of the right-hand tetraloop as drawn (positions 32 and 35) should be transposed; the correct sequence of this loop is ‑C32-T33-T34-G35-. Please note that the sequences shown in Supplementary Table 3 do not suffer this error, and are correct.

Also, in Table 1 of the main paper the average atomic rms deviation from the average structure for the backbone atoms (N, Ca, C) of PARP-1 finger 1 (residues 7-93) should be 0.48 ± 0.11Å (rather than 0.50 ± 0.12Å).

The corrected Fig. 1 and Graphical Abstract appear on next page.

## Figures and Tables

**Fig. 1:**
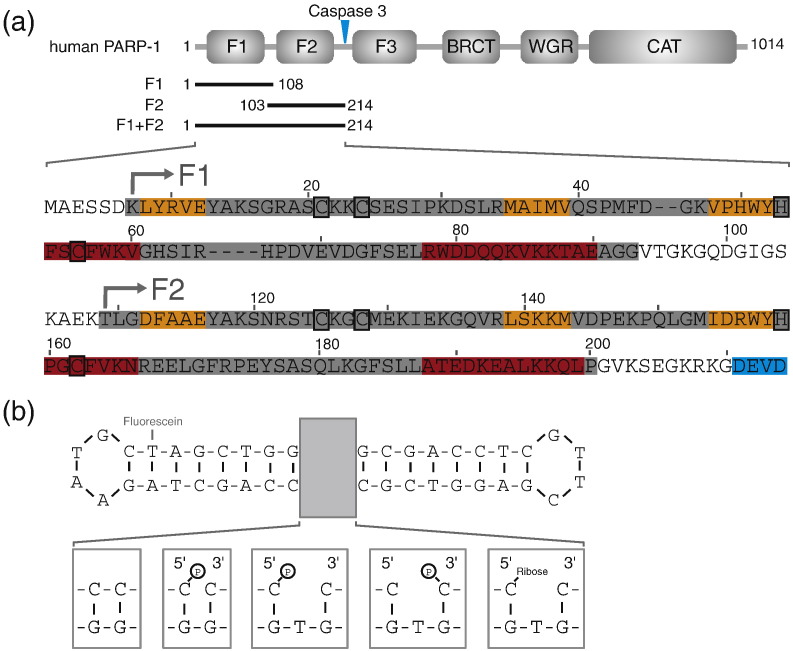


**Graphical Abstract:**