# Supplementary material for: The DNA-Binding Domain of Human PARP-1 Interacts with DNA Single-Strand Breaks as a Monomer through Its Second Zinc Finger
Source: J Mol Biol. 2011 Mar 18;407(1-4):149–70. doi: 10.1016/j.jmb.2011.01.034 (PMC3094755; doi:10.1016/j.jmb.2011.01.034)
Supplement: Supplementary file 1 — Supplementary data [file mmc1.pdf]

Supplementary Material

The DNA-binding domain of human PARP-1 interacts with DNA single-strand breaks as a monomer through its second zinc finger.

Sebastian Eustermann, Hortense Videler, Ji-Chun Yang, Paul T. Cole, Dominika Gruszka, Dimitry Veprintsev and David Neuhaus

MRC Laboratory of Molecular Biology, Hills Road, Cambridge CB2 0QH, U.K.

Supplementary Figures

Supp. Fig. 1 Energy-ordered rmsd profiles and superimposition of F1 and F2 structures

Supp. Fig. 2 Analytical ultracentrifugation sedimentation velocity experiments to analyse the binding of PARP-1 F1+F2 to the 44nt nicked dumbbell DNA ligand

Supp. Fig. 3 Demonstration of slow exchange for PARP-1 F1+F2 interacting with the 45nt gapped DNA dumbbell ligand

Supp. Fig. 4 Fluorescence anisotropy analysis of the interaction between PARP-like zinc fingers and the 44nt nicked DNA dumbbell ligand

Supp. Fig. 5 Different ionic strength dependence of the interaction of PARP-1 F1 and F2 with the 44nt nicked dumbbell DNA ligand

Supp. Fig. 6 [^{15}N-^{1}H] HSQC spectra show that the mutants of PARP-1 F2 maintain their structural integrity.Supp.

Supp. Fig. 7 Electrostatic surfaces of PARP-like zinc fingers

Supplementary Tables

Supp. Table 1 Dissociation Constants

Supp. Table 2 Dissociation Constants of F2 mutants

Supp. Table 3 Sequences of DNA dumbbell ligands

Supplementary Material and Methods

Supplementary References

1) Supplementary Figures

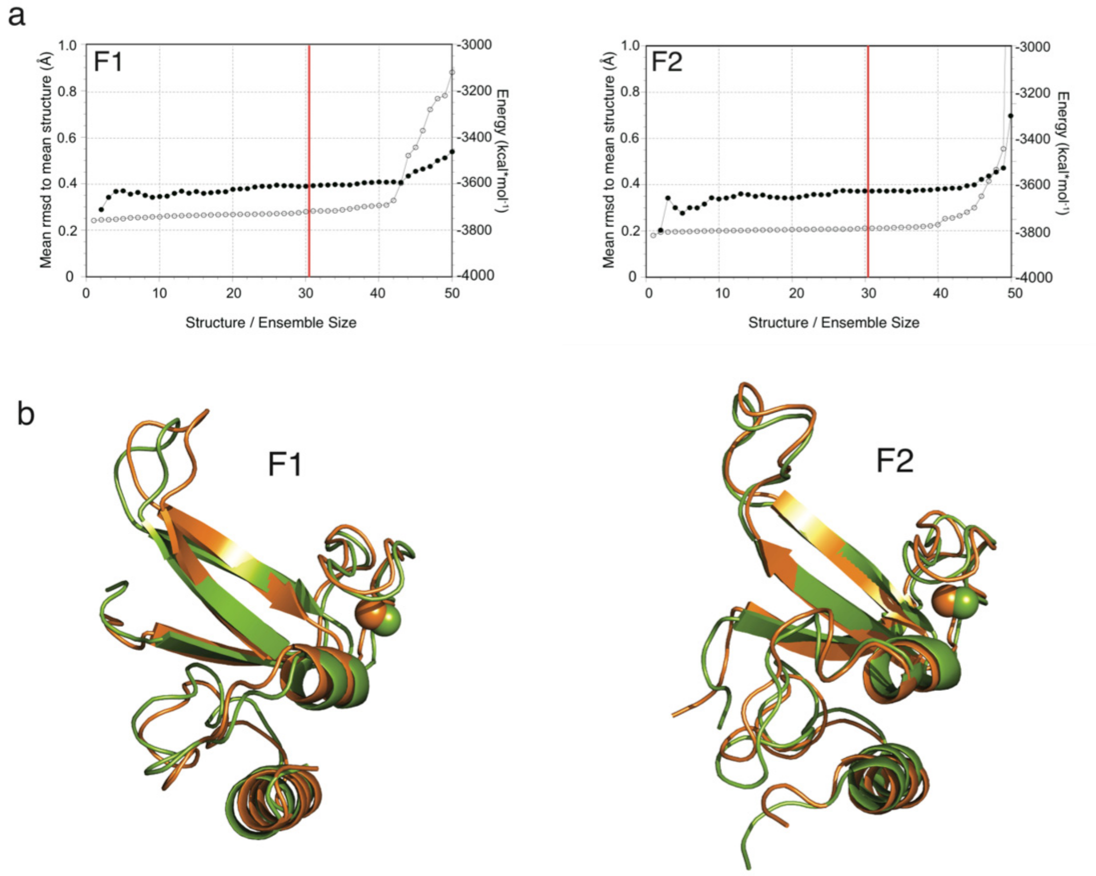

Supplementary Figure 1: Energy-ordered rmsd profiles and superimposition of F1 and F2 structures

(a) Rmsd values (filled circles) are independently calculated using each ensemble size using the program CLUSTERPOSE[1], adding successive structures in order of increasing XPLOR total energy term. Open circles represent the XPLOR total energy terms. Only structures to the left of the vertical red line were included when calculating the structural statistics. (b) Backbone superpositions of the ordered regions of structures from this work (green) with the corresponding structures (orange) determined by the Riken Structural Genomics Initiative (PDB codes 2dmj for F1 and 2cs2 for F2). For F1 the rmsd over residues 7-40 and 46-93 is 1.31Å, while for F2 the rmsd over residues 109-144 and 152-200 is 1.34Å.

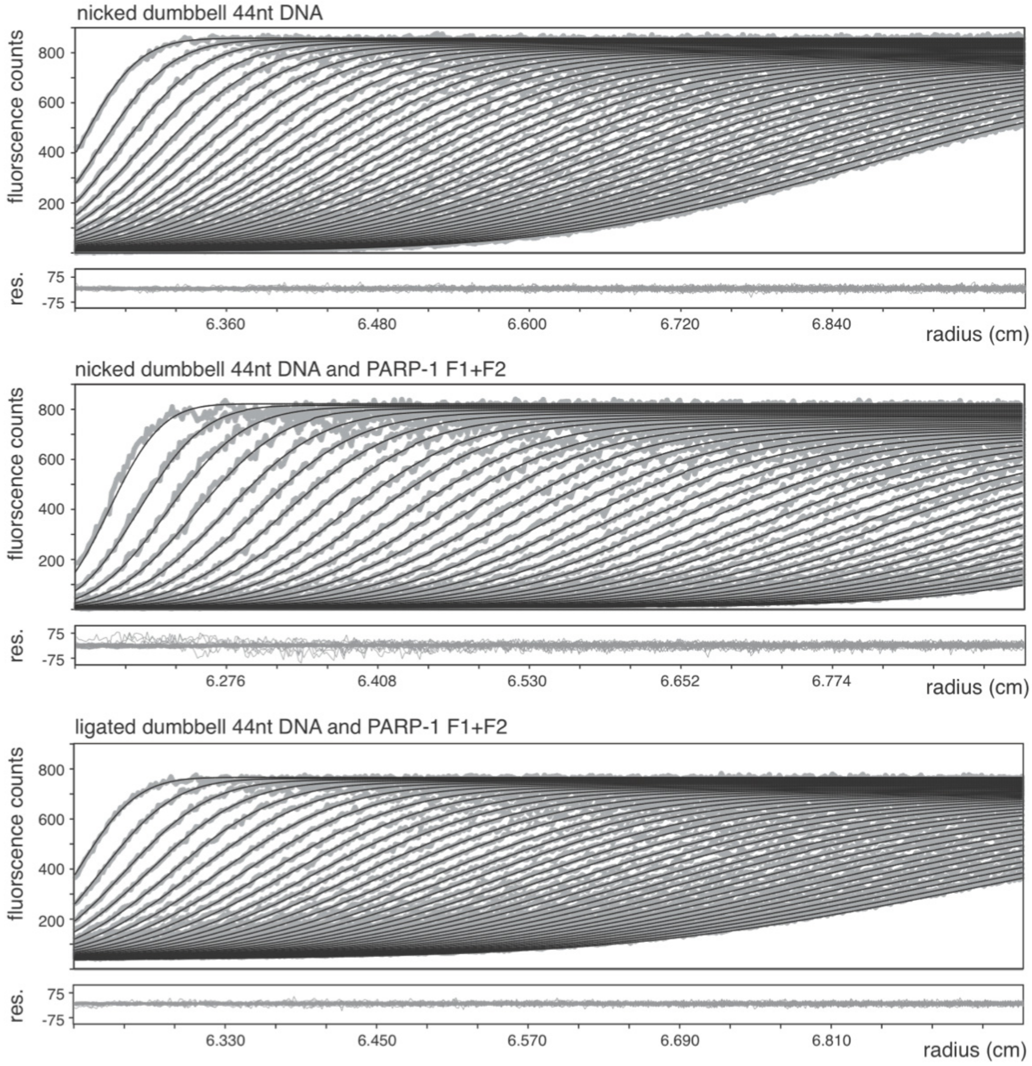

Supplementary Figure 2: Analytical ultracentrifugation sedimentation velocity experiments to analyse the binding of PARP-1 F1+F2 to the 44nt nicked dumbbell DNA ligand.

Raw sedimentation velocity analytical ultracentrifugation data of experiments described in Figure 4. Shown are the DNA concentration versus radius distribution (grey) at different times after start of sedimentation at 45,000rpm. The concentration of fluorescein labeled DNA was measured using a fluorescence detection system. Scans were performed every 78s and every third scan is shown. The program Sedfit[2] was used to fit sedimentation velocity data to the Lamm equation as described in material and methods section. Resulting data fits are shown as black lines and residuals to raw data (res.) are shown below each concentration graph.

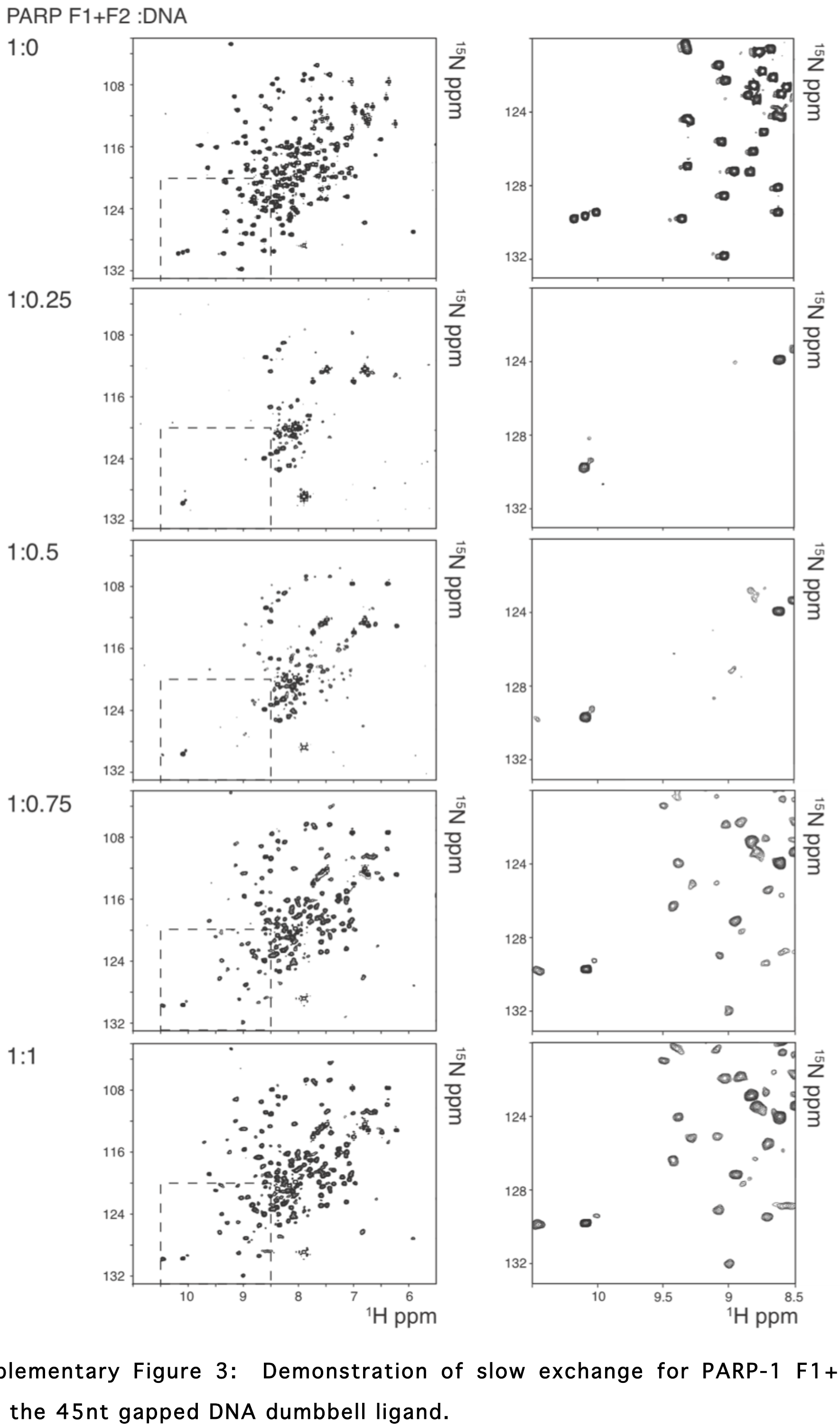

Supplementary Figure 3: Demonstration of slow exchange for PARP-1 F1+F2 interacting with the 45nt gapped DNA dumbbell ligand.

The panels show a titration in which the 45nt gapped DNA dumbbell ligand was progressively added to a sample of PARP-1 F1+F2 (100μM protein in 50mM [$^{2}H_{11}$] Tris pH 7.0, 150μM $ZnSO_4$ and 4mM [$^{2}H_{6}$] DTT). [^{15}N-^{1}H] HSQC spectra at the indicated ratios show that complex formation occurs in the slow exchange regime with respect to the chemical shift timescale, since bound state protein signals appear upon DNA addition with increasing intensity at almost identical peak positions. The initial disappearance of free state protein signals is consistent with the formation of heterogeneous higher order complexes at high molar protein-DNA ratios. Such non-specific DNA binding was also previously observed in fluorescence anisotropy measurements (Figure 4).

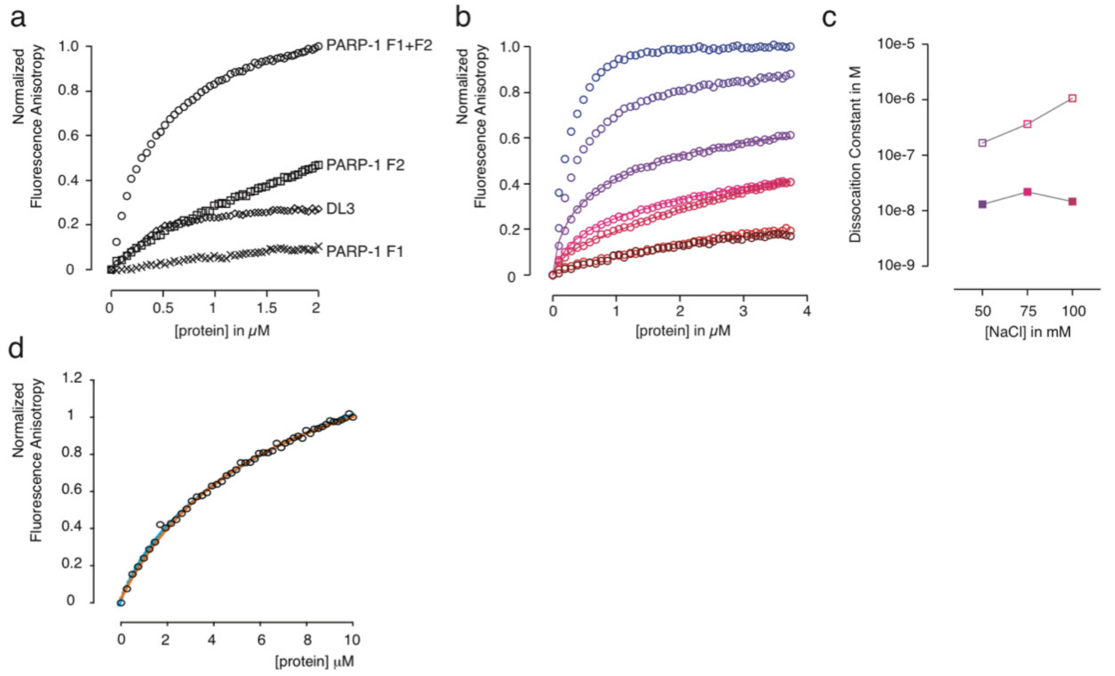

Supplementary Figure 4: Fluorescence anisotropy analysis of the interaction between PARP-like zinc fingers and the 44nt nicked DNA dumbbell ligand.

(a) Fluorescence anisotropy measurements of 44nt fluorescein-labeled nicked DNA dumbbell (10nM) in 20mM pyrophosphate pH 7.0, 4mM DTT and 150μM $ZnSO_4$, titrated with either with PARP-1 F1+F2, PARP-1 F2, PARP-1 F1 or the single zinc finger of DNA Ligase IIIα (DL3; residues 1-117). **(b)** 44nt fluorescein-labeled nicked DNA dumbbell (10nM) in 50mM Tris pH 7.0, 4mM DTT and 150μM $ZnSO_4$ was titrated with PARP-1 F2. Titrations were repeated at different ionic strengths (buffer contained 0mM, 25mM, 50mM, 75mM, 100mM, 150mM, 200mM sodium chloride) and data is colored stepwise from blue to red, accordingly. **(c)** The data shown in (b) has been fitted using a two binding site model as described in the caption of Figure 4. However, a reliable data fit was not obtained for low ionic strength measurements (0 and 25mM), as the fluorescence signal changed by more than 10% during the titration. At ionic strengths of 150mM and 200mM the binding was too weak to be analyzed. **(d)** 44nt Fluorescein-labeled nicked DNA dumbbell (50nM) in 20mM pyrophosphate pH 7.0, 4mM DTT and 150μM $ZnSO_4$ was titrated with PARP-1 F1. Note that the protein and DNA concentrations used are both 5-fold higher than those used in the experiments shown in panel a. It is unclear whether this interaction has a specific component, since neither NMR nor bandshift experiments showed clear evidence for homogeneous complex formation. Consistent with this, the data was fitted either with a one binding site model (K_D=6.6μM; blue curve) or with a two binding site model (K_D1=1.1μM and K_D2 =11.0μM; red curve); see Figure 2 for definition of binding site models).

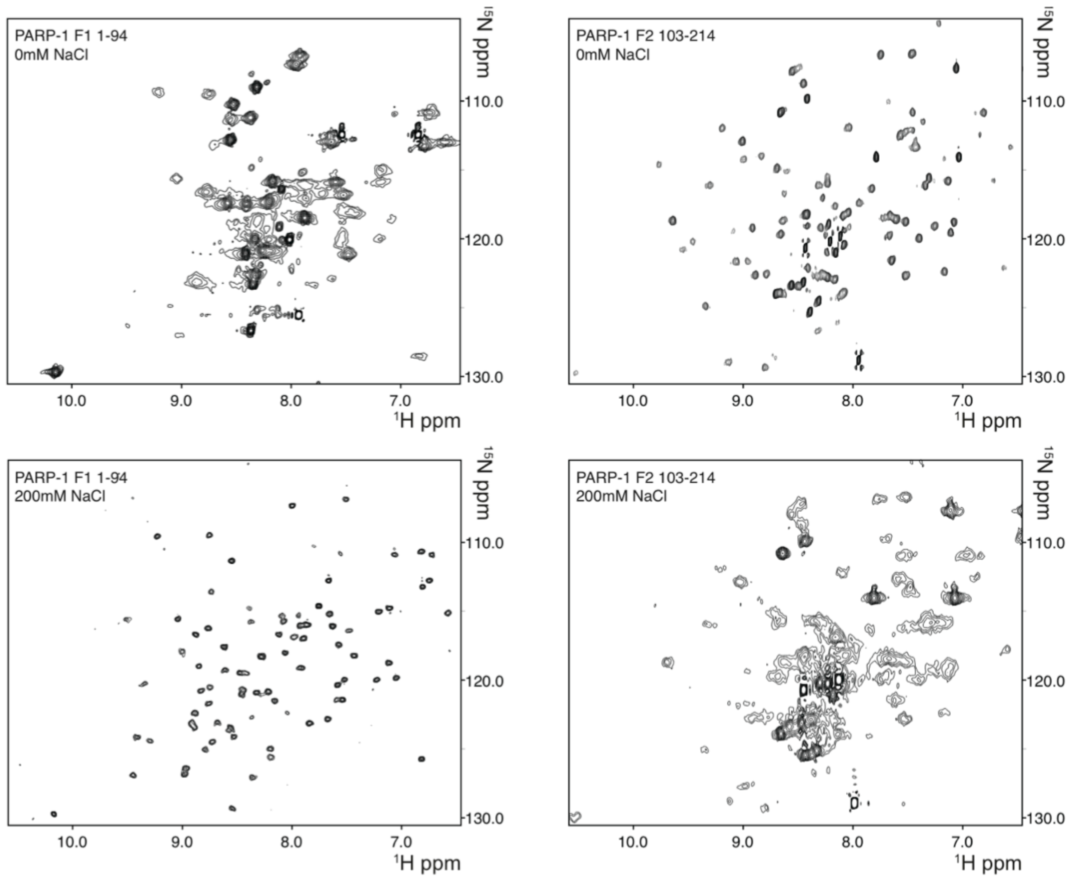

Supplementary Figure 5: Different ionic strength dependence of the interaction of PARP-1 F1 and F2 with the 44nt nicked dumbbell DNA ligand.

PARP-1 F1 (1-94) or PARP-1 F2 (103-214) (100μM protein in 50mM [$^2H_{11}$] Tris pH 7.0, 150μM $ZnSO_4$ and 4mM [$^2H_6$] DTT) were reconstituted in a complex with the 44nt gapped DNA dumbbell ligand either at 0mM NaCl or at 200mM NaCl. At low ionic strength (0mM NaCl), where binding is expected to be strongest, a 1:1 mixture of nicked dumbbell DNA and F2 shows a well-resolved HSQC spectrum with properties consistent with a monodisperse solution of the expected 26kDa complex; this behavior was very similar to that seen for F1+F2 binding to the gapped DNA ligand. In contrast, the HSQC spectrum of a 1:1 mixture of gapped dumbbell DNA with F1 under the same conditions shows many much broader lines, consistent with an exchange process taking place on an intermediate rate on the chemical shift timescale. This exchange could either be between the free and bound states of the protein, if the concentration of free protein under these conditions is sufficiently high to cause appreciable broadening (i.e. > approx. 10%), or it could arise from conformational exchange within a dynamic protein-DNA interface, which would also be consistent with weaker binding (or from a combination of both processes). At higher ionic strength (200mM NaCl), where binding is substantially weaker and off-rates faster, the appearance of the spectra appears at first sight to be reversed between the F1 and F2 cases. However, this is deceptive; in reality, the slow exchange behavior seen at low ionic strength for F2 is shifted to the intermediate regime at higher ionic strength as a result of weakened binding, while the intermediate regime behavior seen for F1 at low ionic strength is now shifted to the fast exchange regime at high ionic strength, again as a result of weakened binding. Consistent with this, the chemical shift perturbations (relative to the free protein) seen in the case of F1 at high ionic strength are very small relative to those seen for F2 at low ionic strength (these being the only two sets of conditions for which the comparison can be made, due to the low quality of spectra in which intermediate rate exchange is evident).

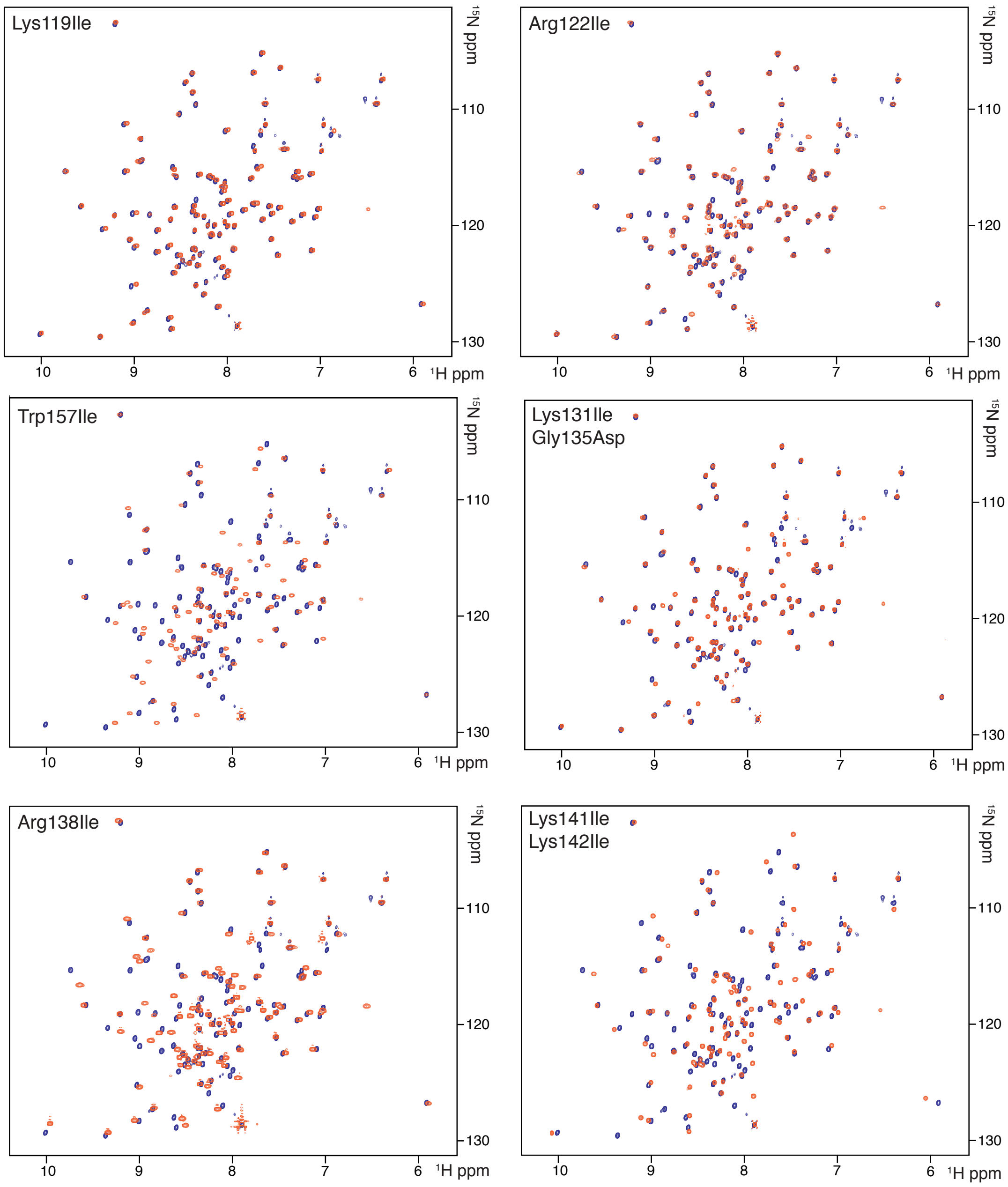

Supplementary Figure 6: [15N-1H] HSQC spectra show that the mutants of PARP-1 F2 maintain their structural integrity.

Each panel shows an overlay of the indicated mutant (red) with the spectrum of wild-type F2 (blue).

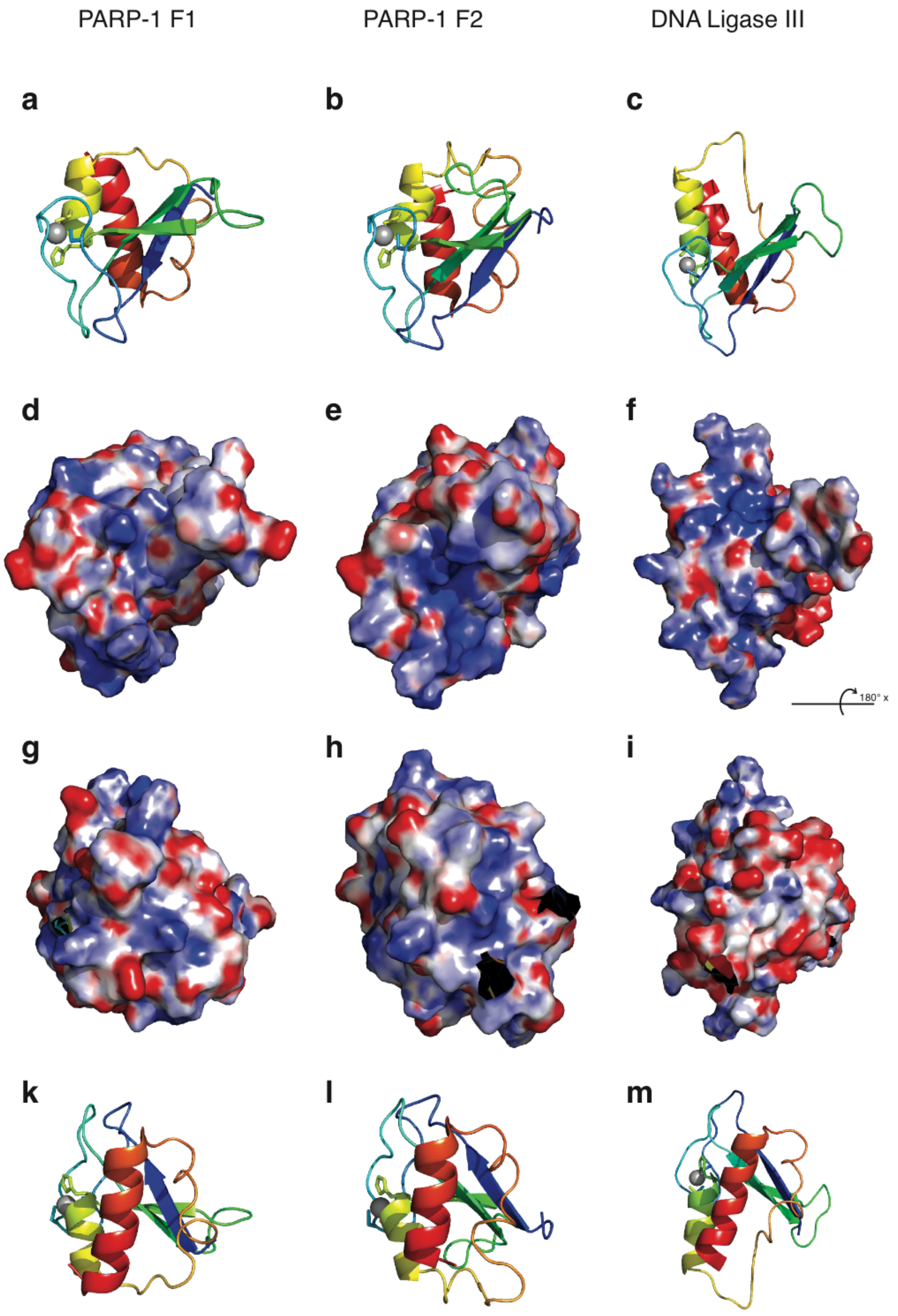

Supplementary Figure 7: Electrostatic surfaces of PARP-like zinc fingers.

Electrostatic potential surfaces are shown for PARP-1 F1, PARP-1 F2 and the single finger of DNA ligase IIIα. In each case, two orientations (related by a 180° rotation about the x axis) are shown, and corresponding cartoon views in chainbow coloring are shown above and below to show the orientation of the structure. Potentials were calculated using the program APBS[3] using a threshold value of ±10eV for the coloring, and visualized using the program pymol.[4] Disordered tails were removed from the display (this is why the surfaces have small missing regions).

The top views **(a-f)** each show the surface of the triple-stranded β-sheet and the long loop L1, which are the principle sites of DNA interaction as mapped by the NMR chemical shift perturbation experiments (see

main text). Both PARP-1 F2 and the DL3 finger have a pronounced basic patch in this part of the surface, although its location differs somewhat between the two. In contrast, the electrostatic surface of PARP-1 F1 is less basic in this region, consistent with its markedly lower affinity for ligands mimicking damaged DNA. The lower views (g-m) show the opposite face of each structure. There are no pronounced basic patches, and in the case of the DL3 finger this part of the surface is predominantly acidic.

Supplementary Tables

Supplementary Table 1: Dissociation Constants

	PARP-1 F1+F2 Nick dumbbell DNA			
NaCl in mM	KD1 in M	Error in M	KD2 in M	Error in M
0	5.75E-09	6.52E-09	1.97E-07	7.01E-08
25	2.04E-08	4.32E-09	4.52E-07	8.82E-08
50	8.02E-09	2.72E-09	7.55E-07	7.88E-08
75	3.70E-08	1.28E-08	1.35E-05	8.32E-05
100	9.37E-09	3.61E-09	2.62E-06	1.15E-06
150	2.46E-08	4.72E-09		
200	4.54E-08	1.10E-08		

	PARP-1 F1+F2 Nick symmetric dumbbell DNA			
NaCl in mM	KD1 in M	Error in M	KD2 in M	Error in M
0	4.86E-08	2.11E-08	3.98E-07	1.83E-07
25	5.42E-08	6.45E-09	1.45E-06	3.24E-07
50	5.61E-08	2.52E-08	7.14E-07	5.46E-07
75	6.24E-08	6.94E-09	7.37E-06	5.16E-06
100	5.69E-08	6.29E-09	6.75E-06	2.99E-06
150	5.24E-08	1.31E-08		
200	4.62E-08	1.50E-08		

	PARP-1 F1+F2 ligated symmetric dumbbell DNA	
NaCl in mM	KD in M	Error in M
0	1.17E-07	4.32E-09
25	1.34E-07	1.84E-09
50	1.66E-07	3.14E-09
75	4.80E-07	2.10E-08
100	1.11E-06	4.18E-08

	PARP-1 F2 Nick dumbbell DNA			
NaCl in mM	KD1 in M	Error in M	KD2 in M	Error in M
50	1.2996E-07	4.0564E-08	1.663E-06	3.5496E-07
75	2.1755E-07	7.1062E-08	3.6336E-06	1.1774E-06
100	1.4593E-07	3.5042E-08	1.0612E-05	2.1731E-06

Supplementary Table 2: Dissociation Constants of F2 mutants

F2	K_D (M) *	Error (M)
WT	3.43E-07	1.50E-08
K119I	4.55E-06	9.16E-08
S120G	2.43E-06	3.75E-08
R122	1.09E-05	3.25E-07
K134I	3.33E-06	6.13E-08
R138I	1.98E-05	9.49E-07
W157I	4.93E-06	7.96E-08
K197I	6.64E-07	1.05E-08
K131I	1.57E-06	2.12E-08
G135D	1.71E-06	3.18E-08
K141I	1.49E-06	3.94E-08
K142I	1.18E-06	1.82E-08
K131I G135D	3.75E-06	1.15E-07
K141I K142I	3.93E-06	1.21E-07

* These apparent K_D values were all obtained using a one binding site model (see text).

Supplementary Table 3: Sequences of DNA dumbbell ligands

	DNA dumbbell ligand sequences*
44nt nick 5'phosporylated	5'-Phosphate-CGGTCGATCGTAAGATCGACCGGCGCTGGAGCTTGCTCCAGCGC
45nt gap 5'phosporylated	5'-Phosphate-CGGTCGATCGTAAGATCGACCGTGCGCTGGAGCTTGCTCCAGCGC-3'
45nt gap 3'phosporylated	5'-CGGTCGATCGTAAGATCGACCGTGCGCTGGAGCTTGCTCCAGCGC-Phosphate-3'
45nt gap 5'ribosylated	5'-deoxyribose-CGGTCGATCGTAAGATCGACCGTGCGCTGGAGCTTGCTCCAGCGC-3'
44nt nick 5'phosporylated fluorescein-dT labelled	5'-Phosphate-CGGTCGA(fluor-dT)CGTAAGATCGACCGGCGCTGGAGCTTGCTCCAGCGC-3'
44nt nick 5'phosporylated fluorescein-dT labelled symmetric sequence	5'-Phosphate-CGGTCGA(fluor-dT)CGTAAGATCGACCGCGGTCGA(fluor-dT)CGTAAGATCGACCG-3'

* the secondary structures and names of the used ligands are given in Figure 1

Supplementary Materials and Methods

Protein Expression:

DNA coding for different fragments of human PARP-1 (F1, residues 1-108, F2, 103-21 and F1+F2, 1-214) was amplified by PCR from the human PARP-1 gene (IMAGE clone 5193735 (Geneservice,UK)) and subcloned into a Pet13 vector (for fragment F1+F2) or a Pet28a vector (for fragment F1 and F2) using BamHI and NcoI restriction sites.

The resulting plasmids were transformed into *E.Coli* BL21-CodonPlus(DE-3)-RP cells (Stratagene). A colony of freshly transformed cells was cultured in M9 minimal medium that contained 50μg/ml Kanamycin as well as 30μg/ml Chloramphenicol and was supplemented either with $^{15}NH_4Cl$ (0.5g/L) or with $^{15}NH_4Cl$ (0.5g/L) and [$^{13}C_6$]-glucose (2g/L) (Sigma Aldrich Isotec) as a sole nitrogen or carbon source, respectively. Cells were grown at 37°C until A_{600} of 0.6 was reached and protein was expressed overnight at 22°C after induction using 0.5mM IPTG. Upon induction the medium was also supplemented with 0.5mM $ZnSO_4$. Protein purification was performed at 4°C throughout. Harvested cells were resuspended in lysis buffer containing 50mM Tris pH 7.4, 150μM $ZnSO_4$, 4mM DTT, 25% (w/v) sucrose and protease inhibitor mix (Roche Complete Protease Inhibitor Cocktail EDTA free; 1 tablet per 50ml). Cell lysis was achieved by sonication, the lysate cleared by centrifugation and filtered using 0.22μM PVDF Stericup filter (Millipore). Initial protein purification carried out via ion-exchange chromatography using SP-Sepharose (GE-Healthcare) eluting with a linear NaCl gradient in 50mM Tris pH 7.4, 150μM $ZnSO_4$, 4mM DTT and protease inhibitor mix (Roche Complete Protease Inhibitor Cocktail EDTA free; 1 tablet per 1L). Eluted protein was exchanged to the same buffer without NaCl and purified further using a HiTrap Heparin HP column (GE-Healthcare) eluting again with a linear NaCl gradient. Finally, protein was purified to homogeneity by size exclusion chromatography using a Superdex-S75 column (GE Healthcare) equilibrated with 50mM Tris pH 7.4, 200mM NaCl, 150μM $ZnSO_4$, 4mM DTT and protease inhibitor mix (Roche Complete Protease Inhibitor Cocktail EDTA free; 1 tablet per 1L).

DNA ligation:

50μM of 5'phosphorylated 44nt nicked DNA was incubated for 16h at 25°C with 2000U T4 Ligase (New England Biolabs) in a total volume of 20μl in a standard ligation buffer (50mM Tris-HCl, 10mM $MgCl_2$, 1mM ATP, 10mM DTT, 25μg/ml BSA) supplemented with 7.5% (w/v) PEG6000. Reaction was stopped by incubating samples for 4min at 65°C. The DNA was purified from T4 Ligase by denaturing polyacrylamide gel electrophoresis as described in Supplementary Reference 5. Completion of ligation was verified by denaturing gel electrophoresis, in which circularized DNA showed a higher mobility than unligated DNA, as described by Ng *et al.*[6]

NMR experiments:

The following spectra were acquired: 2D: [^{15}N-^{1}H] HSQC, [^{13}C-^{1}H] HSQC covering the full ^{13}C spectral width, constant-time [^{13}C-^{1}H] HSQC covering only the aliphatic ^{13}C region, constant-time [^{13}C-^{1}H] HSQC covering only the aromatic ^{13}C region, [^{1}H-^{1}H] NOESY experiments (without heteronuclear filtering; τ_m = 150 ms), [^{1}H-^{1}H] NOESY experiments filtered to remove ^{15}N -coupled signals in F_2 only (τ_m = 150 ms); 3D data sets: CBCANH, CBCACONH, HBHANH, HBHACONH, [^{1}H-^{13}C-^{1}H] HCCH-TOCSY, [^{13}C-^{13}C-^{1}H] HCCH-TOCSY, ^{15}N NOESY-HSQC (τ_m = 150 ms and τ_m = 50 ms), ^{13}C NOESY-HSQC (τ_m = 150 ms), separate datasets acquired for ^{13}C aliphatic and aromatic spectral regions. All of the NOESY datasets used for structure calculations (see below) were acquired using pulse sequences modified to ensure equal RF heating in each case, *e.g.* for ^{13}C experiments, a period of ^{15}N decoupling equal in length to the acquisition period was applied at the beginning of the interscan delay, and for ^{15}N experiments an equivalent period of ^{13}C decoupling was similarly applied.

Residual dipolar couplings (RDCs) were measured using a 0.15 mM solution of ^{15}N labelled PARP-1 F1+F2 adjusted to 50mM [$^{2}H_{11}$] Tris pH 7.0, 430mM NaCl, 150μM $ZnSO_4$, 4mM [$^{2}H_6$] DTT and 5% D_2O (v/v), to which filamentous phage Pf1 (ASLA Biotech) was added to a final concentration of 14mg/ml; splittings were measured in F_1 cross-sections of [^{15}N-^{1}H] HSQC IPAP spectra.[7] Under these conditions a splitting of the D_2O deuterium signal of 14.4 Hz was observed. NOE derived structures of the individual PARP-1 fingers F1 and F2 from XPLOR-NIH calculations were refined using the residual dipolar couplings measured for the two-finger PARP-1 fragment F1+F2 without assuming a fixed alignment tensor by

following the protocol of Sass *et al.*[8] (Note that these structures were not used for deposition; the XPLOR-NIH structures submitted to the final refinement stage in AMBER from which the deposition set was taken were calculated without RDC-derived constraints). The resulting alignment tensors of rigidly defined amide groups ($^{15}N\{^{1}H\}$ heteronuclear NOEs > 0.65) of the structure nearest to the ensemble-average for F1 and similarly for F2 were compared using the program Module 2.0.[9] The uncertainty associated with the magnitude of the of the axial and rhombic component of the alignment tensor were estimated using a Monte-Carlo based error analysis implemented in program Module, setting the measurement error for experimental RDCs as 2Hz.

All spectra were processed using the program TOPSPIN (Bruker GmbH, Karlsruhe) and analysed using either the program SPARKY[10] or CCPN analysis (http://www.ccpn.ac.uk/ccpn).[11]

^{15}N Relaxation Analysis:

$^{15}N\{^{1}H\}$ heteronuclear NOEs as well as longitudinal and transverse ^{15}N relaxation rates for the amide signals of PARP-1 F1+F2 were determined using a 0.45mM solution of a ^{15}N labeled PARP-1 F1+F2 sample adjusted to 50mM [$^{2}H_{11}$] Tris pH 7.0, 200mM NaCl, 150μM $ZnSO_4$, 4mM [$^{2}H_{6}$] DTT and 5% D_2O (v/v) essentially as described by Farrow *et al.*[12] All data were collected at 300K on a Bruker DMX600 spectrometer equipped with a triple resonance ($^{1}H/^{15}N/^{13}C$) cryoprobe. The $^{15}N\{^{1}H\}$ heteronuclear NOE data were collected in an interleaved manner and $^{15}N\{^{1}H\}$ heteronuclear NOE values were calculated from the ratio of peak intensities of pairs of spectra acquired with and without ^{1}H saturation. Longitudinal (T_1) and transverse (T_2) relaxation times of backbone amide protons were determined using T_1 delays of 10, 20, 35, 60, 100, 170, 260, 400, 600, 800 and 1000ms and T_2 delays of 16, 33, 50, 64, 85, 102, 112, 153, 176, 210 and 250ms, respectively. T_1 and T_2 relaxation times were derived from non-linear least square fitting of the measured peak heights to an exponentially decaying function using a modified script of the program Sparky.[10]

Chemical Shift Perturbation Analysis:

^{15}N, ^{13}C-labelled protein samples of PARP-1 F2 and F1+F2 and DNA dumbbell ligands that contained different types of DNA damage (see Results) were adjusted to 50mM [$^{2}H_{11}$] Tris pH 7.0, 150μM $ZnSO_4$, 4mM [$^{2}H_{6}$] DTT and 5% D_2O (v/v). Protein

was titrated stepwise to the DNA up to a 1:1 ratio. The final concentration of the protein-DNA complexes was 100μM and 2D [^{15}N-^{1}H] HSQC were acquired at a temperature of 310K. For protein backbone assignment 1:1 complexes of either F2 or F1+F2 were reconstituted using ^{13}C,^{15}N and (70%)^{2}H labelled protein and unlabelled 45nt gapped DNA dumbbell ligand (200μM complexes in 50mM [$^{2}H_{11}$] Tris pH 7.0, 150μM $ZnSO_4$, 4mM [$^{2}H_6$] DTT and 99% D_2O). TROSY versions of triple resonance HNCA, HNCOCA, HNCACB and HNCOCACB experiments were recorded at 310K using a Bruker DMX 600 spectrometer equipped with a triple resonance (^{1}H/^{15}N/^{13}C) cryoprobe. HNCACB and HNCOCACB experiments were parameterized to obtain primarily Cβ resonances.

^{15}N-labelled PARP-1 F1 was adjusted to 50mM [$^{2}H_{11}$] Tris pH 7.0, 200mM NaCL, 150μM $ZnSO_4$, 4mM [$^{2}H_6$] DTT and 5% D_2O (v/v) and titrated stepwise with 45nt gap 5'phosphorylated DNA dumbbell up to a ratio of 1:1. Since complex formation occurred in a fast chemical shift exchange regime, amide resonance assignment was obtained by following amide chemical shift perturbation upon DNA titration in a series of [^{15}N-^{1}H] HSQC spectra.

Structure Calculations:

Initial structures of PARP-1 fragments F1 and F2 were calculated using the program ATNOSCANDID,[13] for which the input comprises the respective protein sequences (F1 residues 1-108 and F2 residues 103-214), the full resonance assignment and the following 3D NOESY datasets: ^{15}N NOESY-HSQC (τ_m = 150 ms), ^{13}C aliphatic region NOESY-HSQC (τ_m = 150 ms) and ^{13}C aromatic region NOESY-HSQC (τ_m = 150 ms). Dihedral restraints for the backbone were obtained from chemical shifts using the program TALOS.[14] Within ATNOS-CANDID, the internal generation of backbone dihedral restraints was suppressed and the TALOS restraints were specified as external input. During the ATNOSCANDID calculations no metal was represented explicitly, but the effect of metal binding was approximated by including inter-ligand distance constraints as follows: S^{γ} to S^{γ}, 3.7-4.0Å; S^{γ} to histidyl-N, 3.4-3.8Å. At this stage all histidyl N atoms were assigned ambiguously as either N^{δ} or N^{ε} atoms.

In order to be able to employ explicit zinc bonding and geometry terms in the force-field for the calculations (including bond-angle and, for the histidines, in-plane constraints), we next calculated PARP-1 F1 and F2 structures using XPLOR-NIH.[15] As

input, these calculations used the set of NOE restraints generated by the final (seventh) cycle of ATNOSCANDID, re-formatted for use in XPLOR-NIH. Since the XPLOR-NIH calculations employed r^{-6} summation for all groups of equivalent protons and non-stereospecifically assigned prochiral groups, and since no stereoassignments were made (and the assignment-swapping protocol within XPLOR-NIH for deriving stereoassignments indirectly during the structure calculation itself was not applied), the constraints for all such groups were converted to group constraints (*i.e.* such groups were specified using wildcards such as HB*). All lower bounds were set to zero.[16] Structures were calculated from polypeptide chains with randomized phi and psi torsion angles using a two-stage simulated annealing protocol within the program XPLOR-NIH, essentially as described in reference 17.

The structures calculated in XPLOR-NIH were finally subjected to a further stage of refinement using a full force field and an implicit water-solvent model as implemented in the program AMBER 9 (reference). Calculations comprised an initial minimization (200 steps steepest descent then 1800 steps conjugate gradient), then 20ps of molecular dynamics was repeated twice using a simulated annealing protocol (5000x1fs-steps heating from 0K to 500K; 13000x1fs-steps cooling to 100K; 2000x1fs-steps of final cooling to 0.0K) and a final minimization (200 steps steepest descent then 1800 steps conjugate gradient). The experimental distance and TALOS-derived torsional restraints were applied throughout, and force constants for the restraints were increased linearly during the simulated annealing stages to final values of 20 kcal mol^{-1} $Å^{-2}$ for distance restraints and 100 kcal mol^{-1} rad^{-2} for torsion restraints. Implicit solvent representation using the generalized Born method was employed throughout (igb=1), and Langvin temperature control was used (ntt=3; gamma_ln=5).

Fluorescence Anisotropy:

After each addition of protein titrant, the solution was stirred for 30 s, and after 60 s the fluorescence and the fluorescence anisotropy was measured. Data were treated and analyzed essentially as described in Supplementary Reference 18. In the experiments performed, changes in the intensity of fluoresceine fluorescence upon binding of the protein were less than 10% once corrected for dilution. As a result, the fractional fluorescence intensities were assumed to remain constant

throughout the binding titrations and fluorescence anisotropy values were directly fitted to obtain binding affinities. For data analysis fluorescence anisotropy values were normalized between 0 for free DNA and of 1 for the maximal measured value of the respective protein-DNA complex. The program Kaleidagraph (Synergy) was used to obtain a fit of the fluorescence anisotropy data to either a one binding site model $r_{obs}=r_{max}\cdot[P]/(K_D+[P])$ or a two binding site model $r_{obs}=r_{1max}\cdot[P]/(K_{D1}+[P])+r_{2max}\cdot[P]/(K_{D2}+[P])$, in which r_{obs} is the measured and normalized anisotropy value at the given protein concentration [P], r_{max} is the fitted maximal fluorescence anisotropy of the complex species and K_D is the fitted dissociation constant of the interaction. In the two-site model, K_{D1} and r_{1max} are the respective fitted values for the high affinity binding and K_{D2} and r_{2max} are the respective fitted values for the low affinity binding. The variables for fitting were initialized with the following values: $K_D=10^{-6}$, $K_{D1}=10^{-7}$, $K_{D2}=10^{-6}$, $r_{max}=1$, $r_{1max}=0.33$, $r_{2max}=0.66$. Dissociation constants of PARP-1 F2 mutants (Figure 9 and Supplementary Table 2) were derived from fluorescence anisotropy data using a one binding site model. In this analysis r_{1max} was fitted initially for wt PARP-1 F2, while the resulting value ($r_{1max}=1.28$) was used as a constant in subsequent fitting of fluorescence anisotropy data of F2 mutants.

Supplementary References:

1. Diamond, R. (1995). Coordinate-based cluster analysis. *Acta Cryst. D* **51**, 127-135.
2. Schuck, P. (2000). Size-distribution analysis of macromolecules by sedimentation velocity ultracentrifugation and lamm equation modeling. *Biophys J* **78**, 1606-19.
3. Baker, N. A., Sept, D., Joseph, S., Holst, M. J. & McCammon, J. A. (2001). Electrostatics of nanosystems: application to microtubules and the ribosome. *Proc Natl Acad Sci U S A* **98**, 10037-41.
4. DeLano, W. L. (2002). The PyMOL Molecular Graphics System. DeLano Scientific, Palo Alto, CA.
5. Price, S., Oubridge, C., Varani, G. & Nagai, K. (1998). Preparation of RNA:protein complexes for X-ray crystallography and NMR. In *RNA:protein*

interactions : a practical approach (Smith, C. W. J., ed.), pp. 37-72. Oxford University Press, Oxford ; New York.

6. Ng, P. S. & Bergstrom, D. E. (2004). Protein-DNA footprinting by endcapped duplex oligodeoxyribonucleotides. *Nucleic Acids Res* **32**, e107.
7. Ottiger, M., Delaglio, F. & Bax, A. (1998). Measurement of J and dipolar couplings from simplified two-dimensional NMR spectra. *Journal of Magnetic Resonance* **131**, 373-378.
8. Sass, H. J., Musco, G., Stahl, S. J., Wingfield, P. T. & Grzesiek, S. (2001). An easy way to include weak alignment constraints into NMR structure calculations. *Journal of Biomolecular NMR* **21**, 275-80.
9. Dosset, P., Hus, J. C., Marion, D. & Blackledge, M. (2001). A novel interactive tool for rigid-body modeling of multi-domain macromolecules using residual dipolar couplings. *J Biomol NMR* **20**, 223-31.
10. Goddard, T. D., Kneller, D. G. SPARKY 3. University of California, San Francisco.
11. Fogh, R., Ionides, J., Ulrich, E., Boucher, W., Vranken, W., Linge, J. P., Habeck, M., Rieping, W., Bhat, T. N., Westbrook, J., Henrick, K., Gilliland, G., Berman, H., Thornton, J., Nilges, M., Markley, J. & Laue, E. (2002). The CCPN project: an interim report on a data model for the NMR community. *Nat Struct Biol* **9**, 416-8.
12. Farrow, N. A., Muhandiram, R., Singer, A. U., Pascal, S. M., Kay, C. M., Gish, G., Shoelson, S. E., Pawson, T., Forman-Kay, J. D. & Kay, L. E. (1994). Backbone dynamics of a free and phosphopeptide-complexed Src homology 2 domain studied by 15N NMR relaxation. *Biochemistry* **33**, 5984-6003.
13. Herrmann, T., Guntert, P. & Wuthrich, K. (2002). Protein NMR structure determination with automated NOE assignment using the new software CANDID and the torsion angle dynamics algorithm DYANA. *J Mol Biol* **319**, 209-27.
14. Cornilescu, G., Delaglio, F. & Bax, A. (1999). Protein backbone angle restraints from searching a database for chemical shift and sequence homology. *J Biomol NMR* **13**, 289-302.
15. Schwieters, C. D., Kuszewski, J. J., Tjandra, N. & Clore, G. M. (2003). The Xplor-NIH NMR molecular structure determination package. *Journal of Magnetic Resonance* **160**, 65-73.

16. Hommel, U., Harvey, T. S., Driscoll, P. C. & Campbell, I. D. (1992). Human epidermal growth factor: high resolution solution structure and comparison with human transforming growth factor α. *Journal of Molecular Biology* **227**, 271-282.
17. Argentaro, A., Yang, J. C., Chapman, L., Kowalczyk, M. S., Gibbons, R. J., Higgs, D. R., Neuhaus, D. & Rhodes, D. (2007). Structural consequences of disease-causing mutations in the ATRX-DNMT3-DNMT3L (ADD) domain of the chromatin-associated protein ATRX. *Proc Natl Acad Sci U S A* **104**, 11939-44.
18. Weinberg, R. L., Veprintsev, D. B. & Fersht, A. R. (2004). Cooperative binding of tetrameric p53 to DNA. *J Mol Biol* **341**, 1145-59.
